# Recent advancements in the chemistry and therapeutic potential of coumarin motifs

**DOI:** 10.1039/d6ra03929d

**Published:** 2026-10-28

**Authors:** Olayinka O. Ajani, Gbolahan O. Oduselu, Edidiong S. Sam, Mfonobong L. Ekpene, Oluwatosin Y. Audu

**Affiliations:** a Department of Chemistry, Covenant University Km 10, Idiroko Road Ota Nigeria ola.ajani@covenantuniversity.edu.ng +234-8061670254; b Covenant University Bioinformatics Research (CUBRe) Cluster Km 10, Idiroko Road Ota Nigeria; c West African Centre for Cell Biology of Infectious Pathogens (WACCBIP), College of Basic and Applied Sciences, University of Ghana Legon-Accra Ghana; d Holistic Drug Discovery and Development (H3D) Centre, Department of Chemistry, University of Cape Town Rondebosch 7701 South Africa

## Abstract

Coumarin is an oxygen-containing heterocyclic scaffold of considerable importance in medicinal chemistry because of its structural versatility, natural occurrence, and broad range of pharmacological activities. This review provides an integrated overview of the historical development, natural occurrence, physical and chemical properties, synthetic approaches, pharmacological activities, biological targets, structure–activity relationships (SAR), and available toxicological information of coumarin derivatives. Recent literature on coumarin synthesis is examined, with emphasis on Pechmann, Perkin, Knoevenagel, microwave-assisted, metal-catalyzed, and contemporary functionalization strategies. The review also highlights representative clinically established coumarin-based compounds and investigational derivatives, together with their quantitative biological activities, mechanisms of action, and relevant structural features. Reported pharmacological activities include anticoagulant, antimicrobial, antimalarial, antileishmanial, antiviral, antifungal, antioxidant, antidiabetic, antidepressant, anticancer, anti-inflammatory, analgesic, and enzyme-inhibitory activities. Particular attention is given to recurring SAR patterns and biological targets that may guide the rational modification of the coumarin scaffold. Available toxicological and safety data are also considered, highlighting the importance of balancing pharmacological efficacy with therapeutic safety. In addition, the review identifies limitations in the current evidence, including fragmented SAR information, limited mechanistic and *in vivo* validation, and insufficient toxicity data for several investigational derivatives. By integrating synthetic chemistry with pharmacological, mechanistic, SAR, and safety considerations, this review provides a comprehensive perspective on the current progress of coumarin research and highlights opportunities for the development of coumarin-based small-molecule therapeutics.

## Abstract 2

Coumarin is an oxygen-containing heterocyclic scaffold of considerable importance in medicinal chemistry because of its structural versatility, natural occurrence, and broad range of pharmacological activities.

## Graphical abstract

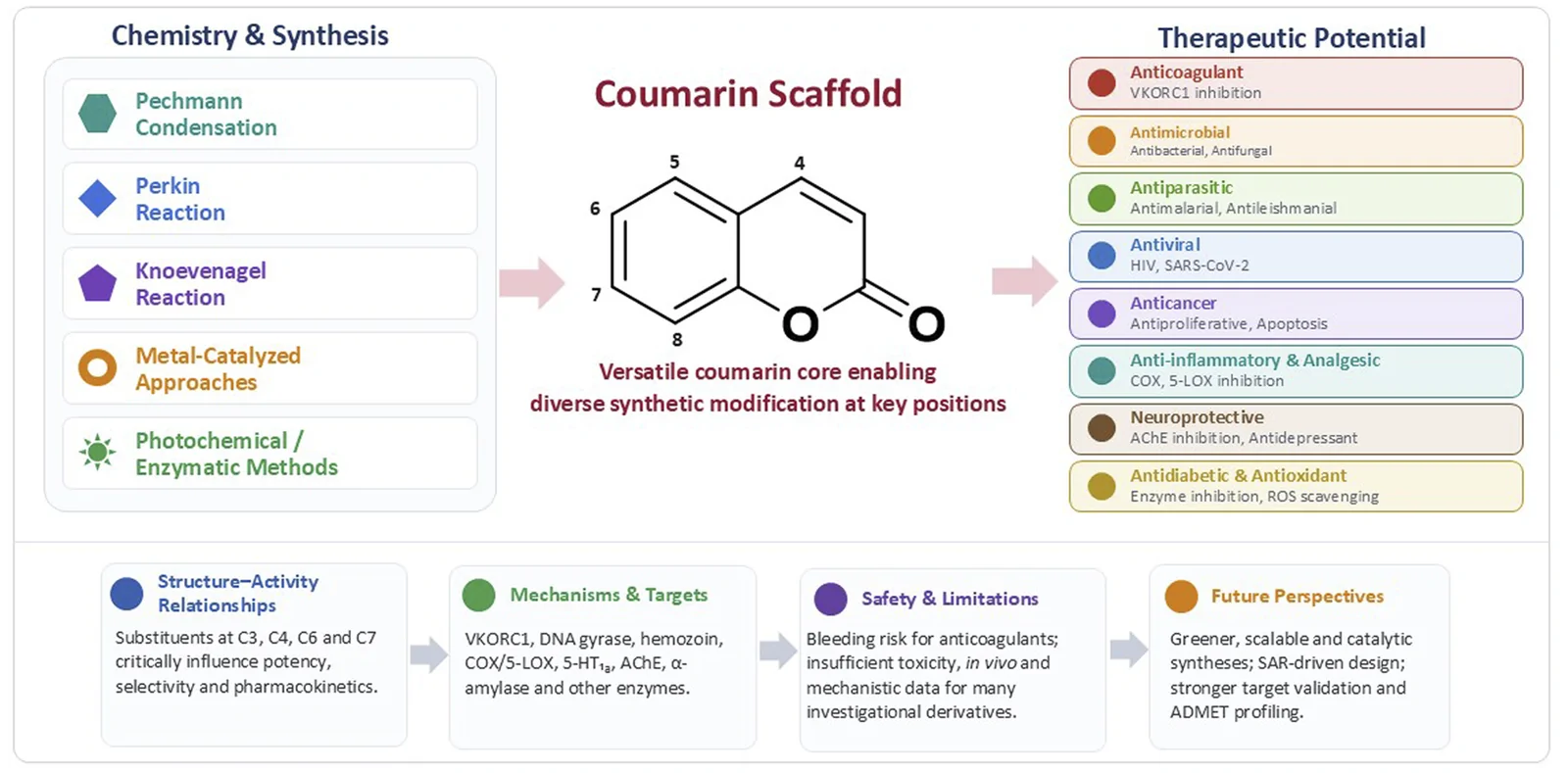

## 1 Introduction

Heterocyclic compounds offer significant biological and pharmaceutical advantages, serving as important structural components of many drugs and biologically active molecules.^1^ The importance of heterocyclic scaffolds in medicinal chemistry and drug discovery is illustrated by recent studies involving diverse nitrogen-, sulfur-, and oxygen-containing heterocycles, including quinoline, spiro-piperidine, 2-aminobenzothiazole, and oxetane scaffolds.^2–5^ Heterocyclic compounds are widely distributed in natural products from plant and animal sources and constitute important structural motifs in naturally occurring organic compounds.^6^ Heterocyclic compounds represent one of the most diverse classes of organic molecules, with wide-ranging chemical, medicinal, and industrial applications.^7^

Heterocyclic compounds can be categorized as aromatic heterocyclic compounds and aliphatic heterocyclic compounds. Cyclic amines, amides, ethers, and thioethers are among the common classes of aliphatic heterocycles. Saturated heterocycles are characterized by the absence of double or triple bonds within the ring system.^8^ Aromatic heterocyclic molecules possess cyclic, conjugated π-electron systems and generally satisfy the requirements for aromaticity described by Hückel's rule, which is commonly expressed as a (4n + 2).^9^

For this review, our focus is on coumarin, an oxygen-containing heterocyclic compound. In organic chemistry, oxygen-containing heterocycles play a significant role. Tetrahydrofuran, coumarin, and oxazole are utilized in various applications, including as solvents, medicines, flavours, and fragrances.^10^ The occurrence of oxygen-containing heterocycles in nature and the variety of their biological roles make them an important class of molecules in organic and medicinal chemistry.^11^ Current therapeutic agents include natural and semi-synthetic oxygen heterocyclic compounds such as lovastatin (hypolipidemic), digoxin (congestive heart failure (CHF) therapy), paclitaxel (Taxol) (anticancer), and cyclosporine-A (immunosuppressant).^12^

Oxygen-containing heterocyclic compounds can be categorized based on the number of oxygen atoms residing in their ring structure. These include fused heterocyclic compounds containing a single oxygen atom such as benzofuran, coumarin, chromene, chromane, and xanthene, and fused heterocyclic compounds containing two oxygen atoms, such as 2,3-dihydro-1,4-benzodioxine, dibenzo-p- dioxine, and 1,4-benzo-dioxine. Heterocyclic compounds containing oxygen can be further classified based on their degree of saturation, aromaticity, and abundance.^13^

Among oxygen-containing heterocycles, coumarin has attracted considerable attention because of its structural versatility, natural occurrence, and broad range of pharmacological activities. The coumarin scaffold is found in numerous natural products and has served as a valuable structural framework in medicinal chemistry. Coumarin derivatives have been investigated for diverse biological activities, including anticoagulant, antimicrobial, antimalarial, antileishmanial, antiviral, anticancer, anti-inflammatory, antioxidant, antidiabetic, neuropharmacological, and enzyme-inhibitory effects.^14–17^

Several reviews have examined different aspects of coumarin chemistry and pharmacology. Hussain *et al.* (2019) reviewed natural-product coumarins and their biological and pharmacological perspectives,^18^ while Garg *et al.* (2020) focused on the therapeutic applications of coumarin compounds and their mechanisms of action.^19^ Jumal and Sakinah (2021) provided an overview of the synthesis, characterization, and applications of coumarin derivatives,^20^ whereas Mustafa (2021) and Sharma *et al.* (2022) focused mainly on classical and recent synthetic approaches, respectively.^21,22^ Other reviews have addressed specific pharmacological areas, including the potential antidiabetic effects of coumarin-related compounds and the anticancer activities of coumarin derivatives.^23,24^

Accordingly, the present review provides an integrated overview of literature on coumarin derivatives published from 2014 to 2026, covering their historical development, natural occurrence, physical and chemical properties, classical and contemporary synthetic approaches, pharmacological activities, biological targets and mechanisms of action, SAR trends, and available toxicological information. Earlier seminal studies are included where necessary to provide historical and mechanistic context. Particular emphasis is placed on representative coumarin-based drugs and investigational compounds, quantitative pharmacological data, comparative analysis of synthetic methodologies, and recurring structure–activity relationships across different biological activity classes. By bringing these aspects together, this review aims to provide a comprehensive perspective on the current state of coumarin research, identify limitations in the existing evidence, and highlight opportunities for future medicinal chemistry and drug-development studies.

## 2. Historical background on coumarin

Coumarins (2*H-l*-benzopyran-2-ones) are oxygen-containing heterocycles commonly used in pharmaceuticals and dyes (Fig. 1). The name coumarins was derived from the French word “tonka bean” (dipteryx odorata Wild., Fabaceae), from which coumarin was discovered in 1820. They are a group of lactones with a benzopyrone structure that have been isolated from plants and fully synthesized in the lab. The parent coumarin's properties are altered by the incorporation group, which is fused with it to create a more valuable product.^25^ Coumarin and its derivatives are a naturally occurring class of benzopyrones with their single-bonded oxygen heteroatom occupying position 1. They are a significant class of naturally occurring and synthetic heterocycles containing oxygen, characterized by a benzopyrone framework.

**Fig. 1:**
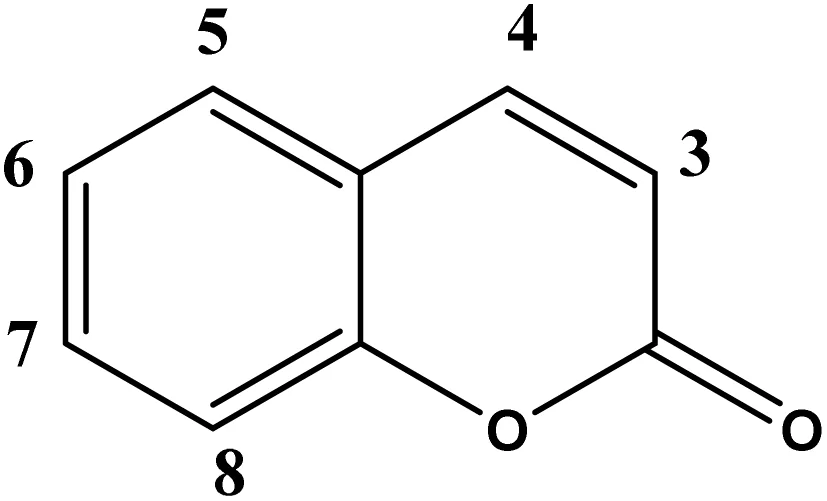
Coumarin's chemical structure and the IUPAC number for this scaffold.

Coumarins have demonstrated considerable relevance to medicinal chemistry, with several coumarin-based compounds achieving therapeutic or clinical significance. The discovery of dicoumarol as an anticoagulant established an important medicinal application of the coumarin scaffold, subsequently leading to the development and widespread clinical use of vitamin K antagonists such as warfarin, acenocoumarol, and phenprocoumon. In addition to anticoagulants, the aminocoumarin antibiotic novobiocin provides another important example of the therapeutic utility of the coumarin framework.^26^ These compounds illustrate how structural modification of the coumarin scaffold can generate diverse pharmacological properties and therapeutic applications.

## 3. Natural occurrence of coumarin

Coumarins and their derivatives are a significant class of natural chemicals widely distributed throughout the natural world.^27^ The fruits and flowers have the largest concentration, although they also exist in the integument of leaves, fruits, stems, roots, seeds, and flowers. *Dipteryx odorata* seed (bean seed) was used for the first time to isolate coumarin. Secondary metabolites called coumarins are found in sponges, bacteria, fungi, and higher plants. This type of secondary metabolite serves as a defence mechanism against herbivores and microbial attacks. The shikimic acid pathway is used to produce these compounds from phenylalanine. Unsaturated lactones from the C6C3 chemical class make up natural coumarins. The oxygenated substituent at position 7 of almost all naturally occurring coumarins can be mixed as in methyl, sugars, *etc*, or free (hydroxylated umbelliferon), in various derivatives. They fall into the structural category of *ortho*-hydroxycinnamic acid derivatives.^28^

## 4. Physical properties of coumarin

The coumarin ring is formed when a benzene molecule and a pyrone ring fuse. Many plant species contain coumarin derivatives, which typically occur as secondary metabolites. Coumarin is a volatile, white crystalline substance, as indicated by its physical characteristics. It has a vanilla-like odour and a bitter taste, with the molecular formula C_9_H_6_O_2_. Coumarin is soluble in ethanol; very soluble in ether and chloroform, with a 71 °C melting point and a 297–299 °C boiling point. The molecular mass is 145 g mol^−1^, with a density of 0.94 g cm^−3^.

## 5. Various chemical reactions of coumarins

### 5.1. Metal-catalyzed reactions

In the production of coumarins and their derivatives, the use of metals as catalysts has become a crucial tool. The most popular metal engaged in the heterogeneous catalytic synthetic approach towards the production of diverse coumarin motifs is palladium; others include cobalt, iron, silver, platinum, zinc, gold, rhodium, gallium, nickel, and copper.^29^

Bhatia and his colleagues published a paper in which numerous synthetic reactions, including intramolecular hydro arylation, Buchwald–Hartwig coupling, Pechmann condensation, C–H alkenylation, oxidative cyclocarbonylation, oxidative Heck coupling, dicarbonylation, site-selective cross-coupling, carbonylative annulations, oxidative annulations, and cycloaddition reaction, among others, were catalyzed by metals (Scheme 1). By boosting reaction rates and product yields, these metal-catalyzed methods for building coumarin rings significantly expand the applicability of conventional techniques.^30^

**Scheme 1:**
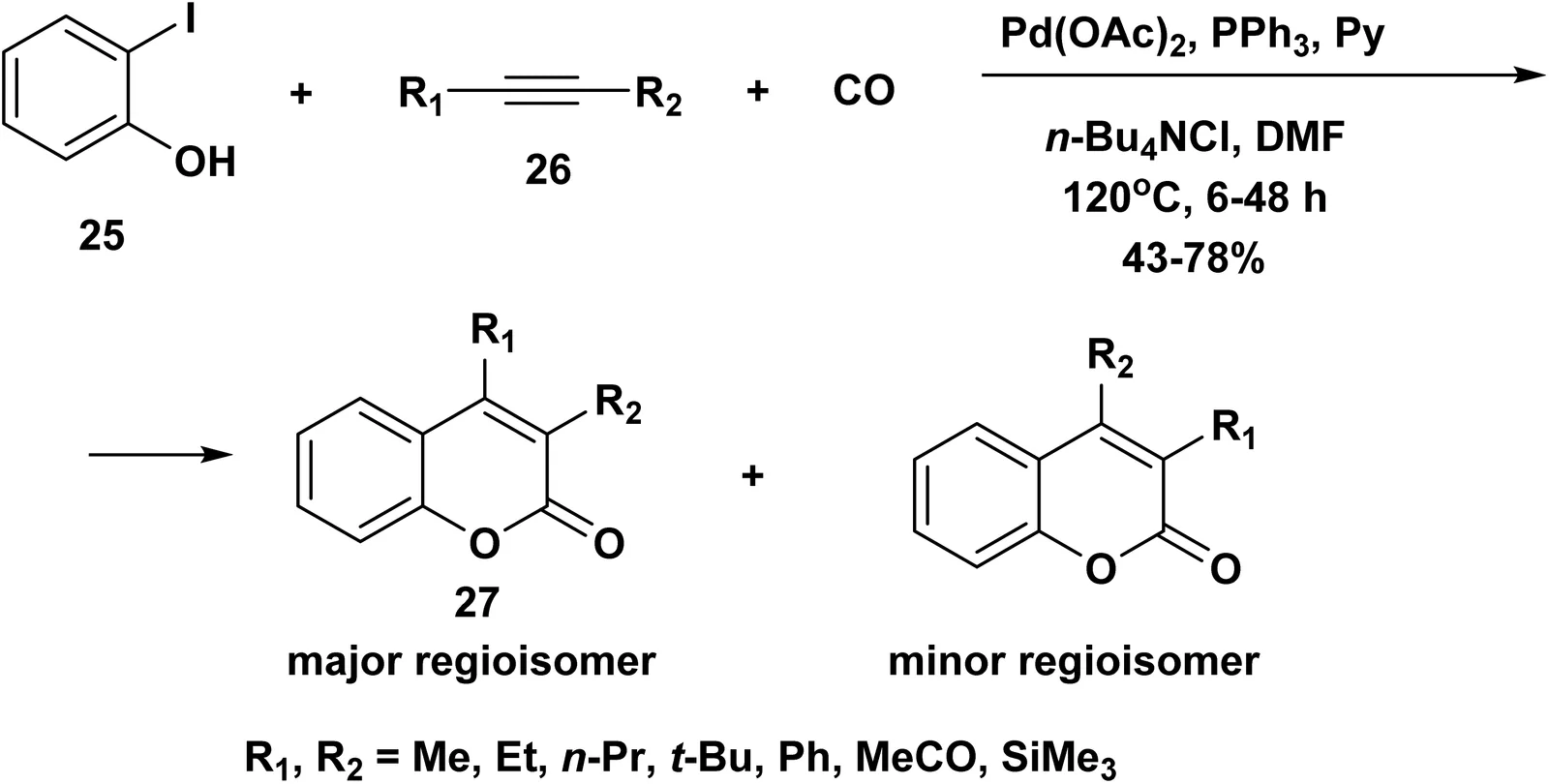
Metal-catalyzed reactions of coumarin compounds.

### 5.2. Pechmann condensation reaction

The Pechmann condensation is a widely used method for the synthesis of coumarin derivatives because it employs readily available starting materials and can provide high yields under relatively mild conditions.^31^ The reaction typically involves the acid-catalyzed condensation of phenols with β-ketoesters to form coumarin derivatives. Various Lewis acids, including GaI_3_, ZrCl_4_, InCl_3_, and Sm(NO_3_)_3_, have been investigated as catalysts for this transformation.^32^ Karami and Kiani employed ZrOCl_2_·8H_2_O supported on SiO_2_ as a heterogeneous catalyst for the Pechmann condensation of substituted phenols with β-ketoesters under solvent-free conditions (Scheme 2). Using 10 mol% of ZrOCl_2_·8H_2_O/SiO_2_ at 90 °C, the reaction afforded the corresponding coumarins in 75–99% isolated yields within 5–80 min. For example, the reaction of resorcinol with ethyl acetoacetate afforded coumarin in 94% yield after 40 min, while several other substrates afforded products in 89–99% yields. Phloroglucinol derivatives showed particularly high reactivity, with products obtained in 98% yield. The authors also reported that the catalyst could be recovered and reused, highlighting the potential of the ZrOCl_2_·8H_2_O/SiO_2_ system as a practical heterogeneous catalyst for Pechmann coumarin synthesis.^33^

**Scheme 2:**
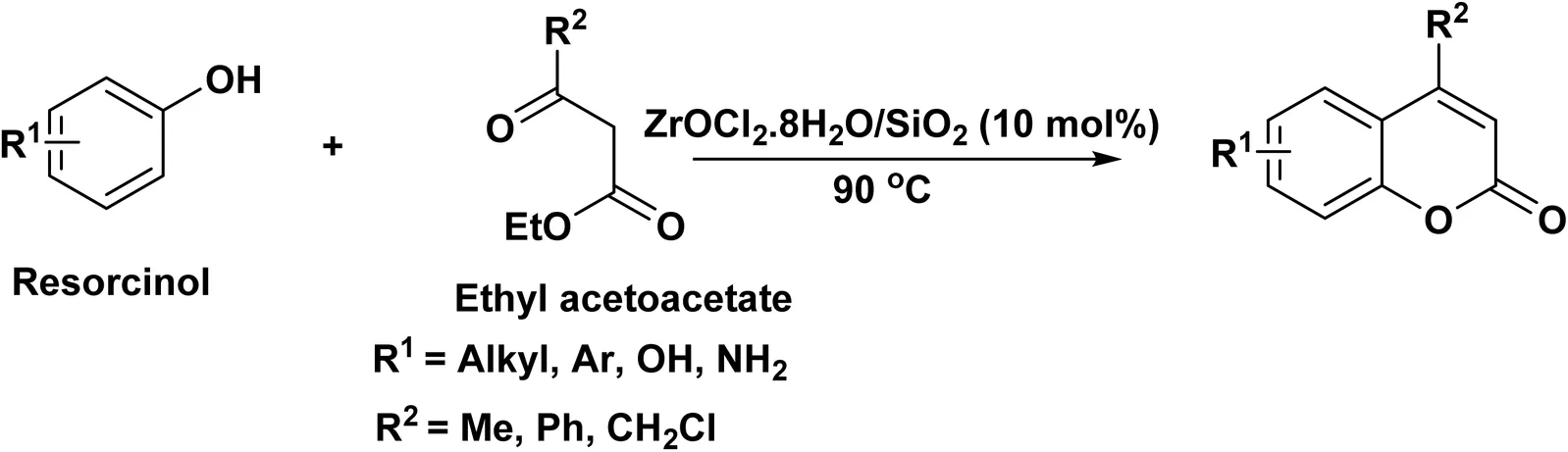
Coumarin synthesis by Pechmann condensation.

### 5.3. Perkin reaction

The first instance of this type of condensation reaction was described by Perkin in 1868, involving the combination of coumarins through the condensation of the sodium or potassium salt of salicylaldehyde with acetic anhydride.^34^

When an aromatic aldehyde and an acid anhydride are combined in the presence of an alkali salt of the acid, the Perkin reaction occurs, an organic process used to create an unsaturated aromatic acid. Other bases may be utilized in place of the alkali salt, which serves as a base catalyst. A substituted coumarin is created after an intramolecular cyclization. *O*-hydroxybenzaldehyde condensation is a crucial step in the creation of coumarins. However, when a mixture of trimethylamine, 2-thiophene acetic acid, 2-fluorobenzaldehyde, and acetic anhydride is heated, coumarins instead of the anticipated cinnamic acid are produced (Scheme 3).^34^

**Scheme 3:**
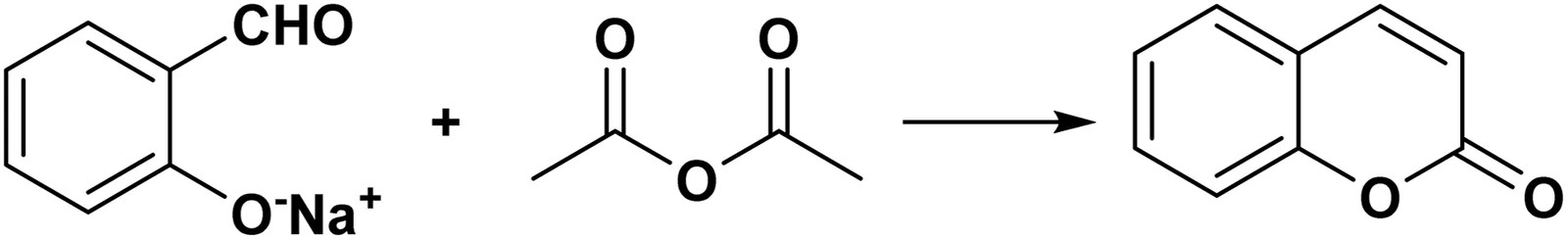
Perkin reaction of coumarin.

### 5.4. Microwave-promoted reactions

Organic synthesis using microwaves has recently overshadowed traditional heating techniques. The use of microwave energy to heat chemical reactions has become increasingly important in recent years for the synthesis of organic molecules. Microwave irradiation reduces by-product formation, increases yield, and enhances reproducibility, while dramatically accelerating chemical reactions. In order to effectively synthesize and find new chemical substances, several synthetic chemists have attempted to adopt microwave heating as a technique for quick reactions.^35^ According to reports, a Pechmann microwave-assisted reaction can be used to quickly, easily, and affordably synthesize compounds that are derived from coumarin. For instance, coupling ethyl acetoacetate with 3-cresol in the presence of a microwave enabled the synthesis of 4,7-dimethylcoumarin, as shown in Scheme 4.^36^

**Scheme 4:**
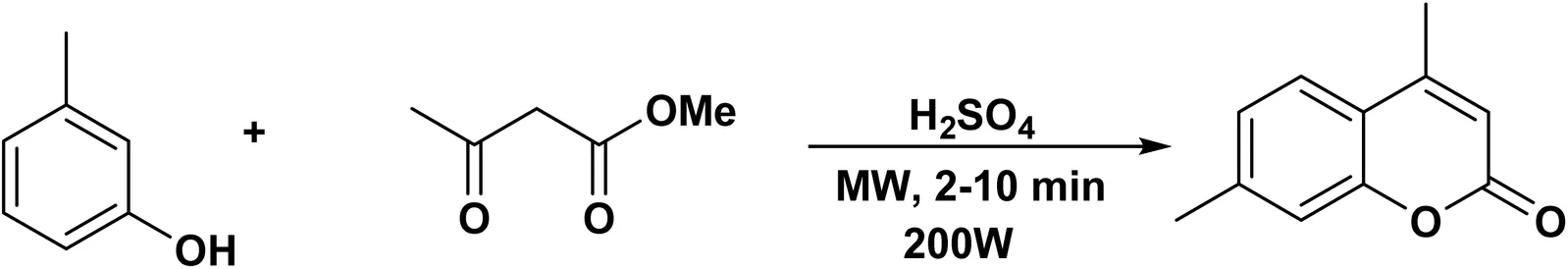
Microwave-mediated reaction to give a coumarin compound.

### 5.5. Knoevenagel reaction

Knoevenagel reaction entails the base-catalysed condensation of an aldehyde or ketone carbonyl with an ester bearing an α-hydrogen from an active methylene. The catalyst is usually a sterically hindered amine or a weak base secondary amine like piperidine, while the α-hydrogen should have functional moieties such as carboxylate, carbonyl (of either aldehyde or ketone), CN, or NO_2_. Typically, aprotic organic solvents such as DMF, MeCN, or pyridine, which serve as both catalysts and solvents, are used to conduct the reaction.^37^

Bases can facilitate the synthesis of 3-substituted coumarin derivatives. The base must be present, the reaction must be heated, and there must be 2-hydroxybenzaldehyde and a coupling partner with an active methylene group present. Scheme 5 depicts the proposed mechanism for this reaction. There are several reports in the literature on the synthesis of coumarin scaffolds *via* the Knoevenagel reaction under solvent-free conditions and under ultrasound.^38^

**Scheme 5:**
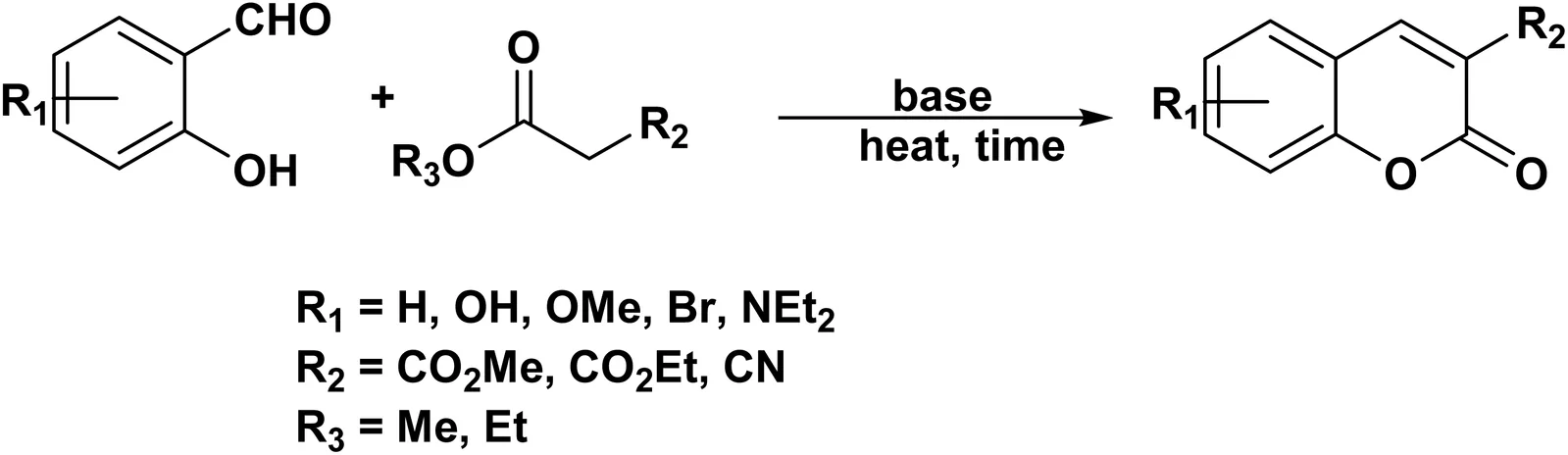
Synthesis *via* Knoevenagel reaction.

## 6. Synthesis of coumarin derivatives

The synthesis of coumarin derivatives has attracted considerable interest because of the importance of the coumarin scaffold as a pharmacophore. Numerous synthetic approaches have been developed using different starting materials and reaction conditions. This article summarizes recent studies that employed various components, techniques, and procedures in the synthesis of coumarin derivatives.

### 6.1. Synthesis of 3-phosphorylated coumarins using silver as a catalyst

Yuan and co-workers developed an effective and selective method for the phosphorylation of the C-3 position of the coumarin nucleus using AgNO_3_ in the presence of Mg(NO_3_)_2_·6H_2_O. The method involved direct C(sp^2^)–H radical phosphorylation of coumarins with H-phosphites. The effects of different H-phosphites and reaction conditions were subsequently investigated. Under the optimized conditions, various dialkyl H-phosphites reacted with coumarin to afford the corresponding 3-phosphorylated derivatives in moderate to good yields (45–65%) (Scheme 6). Diisopropyl and di-*sec*-butyl H-phosphites afforded yields of 56% and 59%, respectively, while the optimized model reaction gave a 60% yield. The method provided selective access to 3-phosphorylated coumarins under mild reaction conditions.^39^

**Scheme 6:**
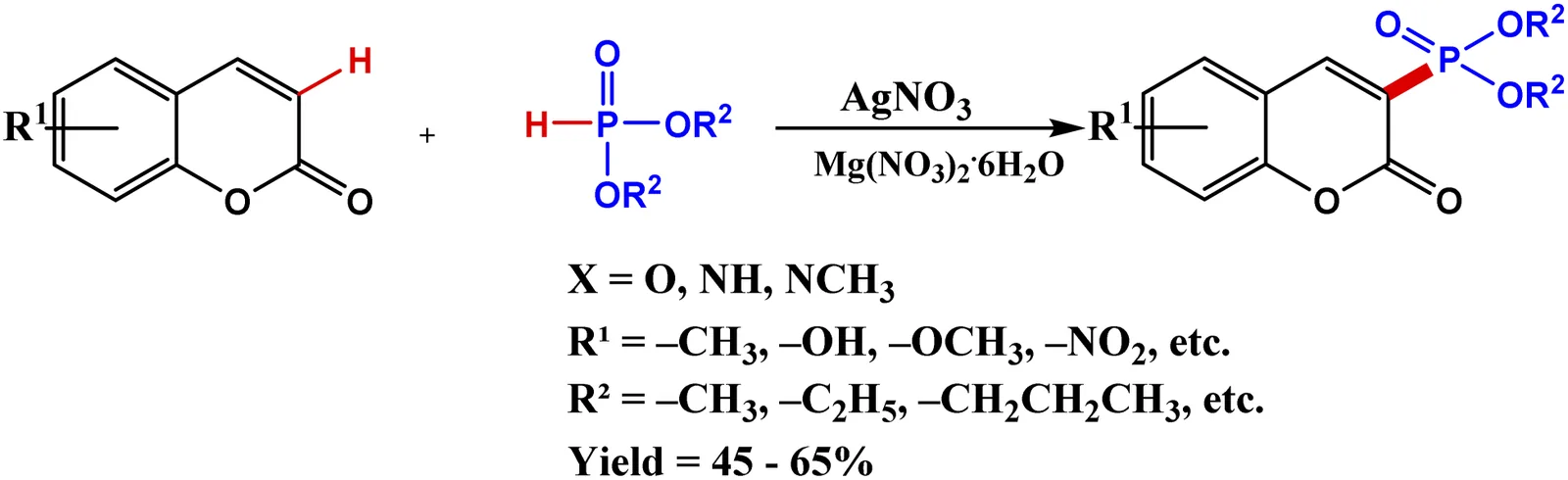
Silver-catalyzed synthesis of 3-phosphorylated coumarins.

### 6.2. Synthesis of indolo[2,3-*c*] coumarins and indolo[2,3-*c*] quinolinones

Vaillard and co-workers previously reported a photoinduced C–H/C–X coupling of 2-iodophenyl-1*H*-indole-2-carboxylate in liquid ammonia using *t*-BuOK as a base, affording the corresponding indolo[2,3-*c*]coumarin in yields of up to 93% (Scheme 7).^40^ Vaillard's approach, as well as other previous approaches, concentrated on the synthesis of the coumarin lactone ring in isoazacoumestans, which frequently required multi-step conversions to reach the pre-functionalized substrates and, at times, harsh reaction conditions.

**Scheme 7:**
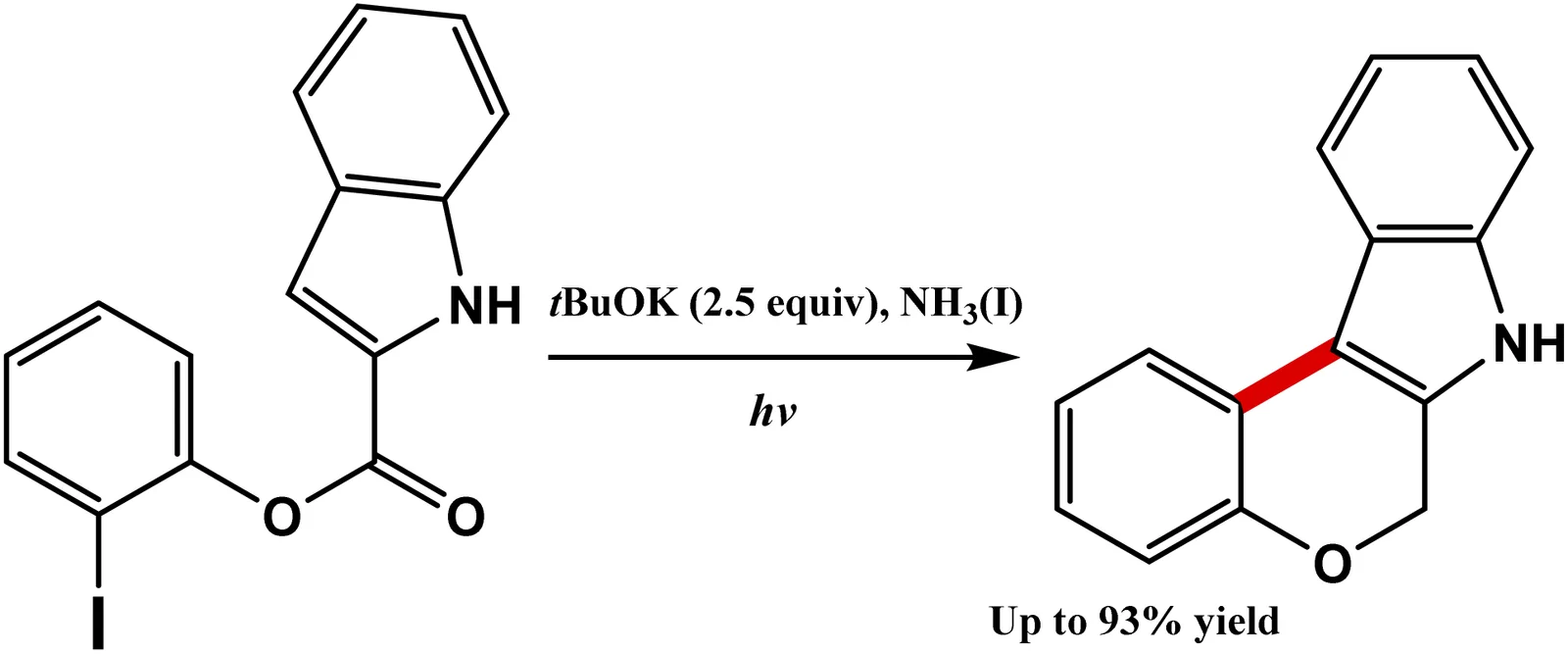
Photoinduced synthesis of an indolo[2,3-*c*]coumarin *via* C–H/C–X coupling of 2-iodophenyl-1*H*-indole-2-carboxylate using *t*-BuOK in liquid ammonia.

Gu and co-authors then utilized a microwave-irradiated approach to drive intramolecular cyclization of 3-anilino-coumarin to yield a tetracyclic coumarin. This was more effectively accessed in a non-basified environment.^41^ This method is a simple and convenient means to obtain a broad range of indolo[2,3-*c*] coumarins and indolo [2,3-*c*] quinolinones from readily available 3-arylamino coumarins with the use of Pd-catalyzed intramolecular cross-dehydrogenative coupling (CDC) reactions (Scheme 8). This method is characterized by great atom conservation and good functional group compatibility.^42^

**Scheme 8:**
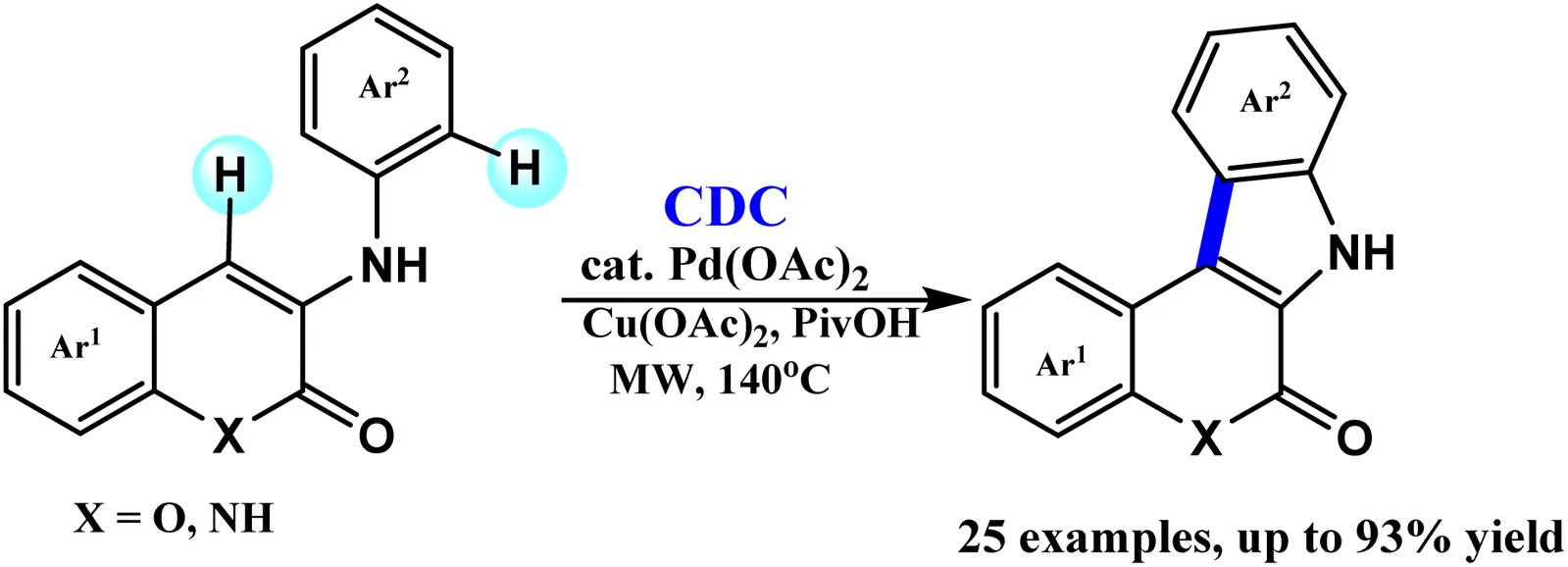
Synthesis of indolo[2,3-*c*] coumarins.

### 6.3. Catalyst-free arylsulfonylation cyclization of phenyl 3-phenylpropiolates

Ren and co-authors^43^ established a simple and effective method for aryl sulfonyl incorporation in the synthesis of 3-sulfonyl coumarins, using ipso-cyclization and potassium persulfate in an acetonitrile medium. Previously, the orthocyclization mechanism involving radical intermediates was used to produce coumarin with substituents in position 3 *via* phenyl 3-phenylpropiolates (Scheme 9). Later, for these changes, an ipso-cycling protocol was postulated and verified by experimental data. Ren and his co-workers devised a new, effective, straightforward method for the cyclization in an eco-friendly manner, which is without a catalyst, affording the 3-sulfonyl coumarins in yields of up to 93%.^44^

**Scheme 9:**
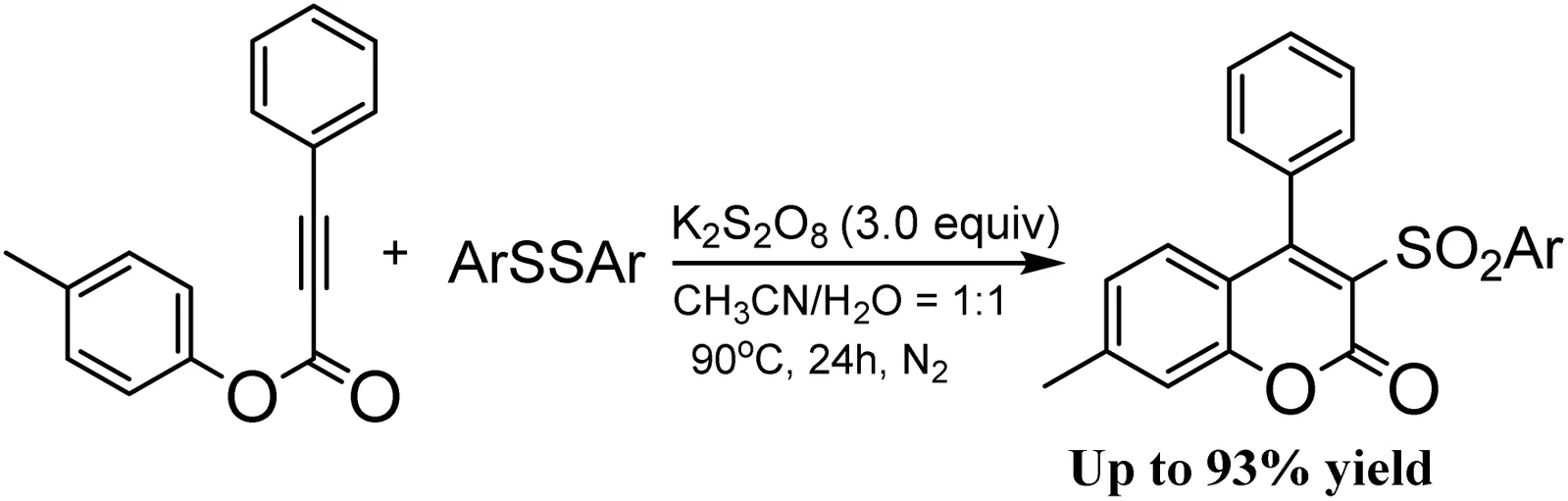
Methods for the synthesis of 3-sulfonyl coumarins.

### 6.4. Synthesis of coumarin from the photoinduced cyclization of alkynoates

Lei wang and his team^45^ devised an effective photoinduced technique for coumarin production. A photoinduced intramolecular cyclization and ester rearrangement provide an alternate, easy route *via* equivalent alkynoates. A wide range of substrates was used under ambient conditions to obtain the products in excellent yields. Under the optimized conditions, the model reaction afforded the desired coumarin in 95% isolated yield, while representative substituted substrates gave yields ranging from 51% to 89%.^46^ Some iodine sources such as Iodobenzene, tetrabutylammonium iodide (TBAI), I_2_, and *N*-iodosuccinimide (NIS) were evaluated during an optimization study to ascertain their role in a yield-enhancing situation (Scheme 10). In their final result, they found *N*-iodosuccinimide to be the best iodine source. This approach uses visible light to drive synthesis *via* electron transfer. It is a simple, effective, and metal-free process for synthesizing coumarins with wide functional-group tolerance under moderate, environmentally favourable conditions.

**Scheme 10:**
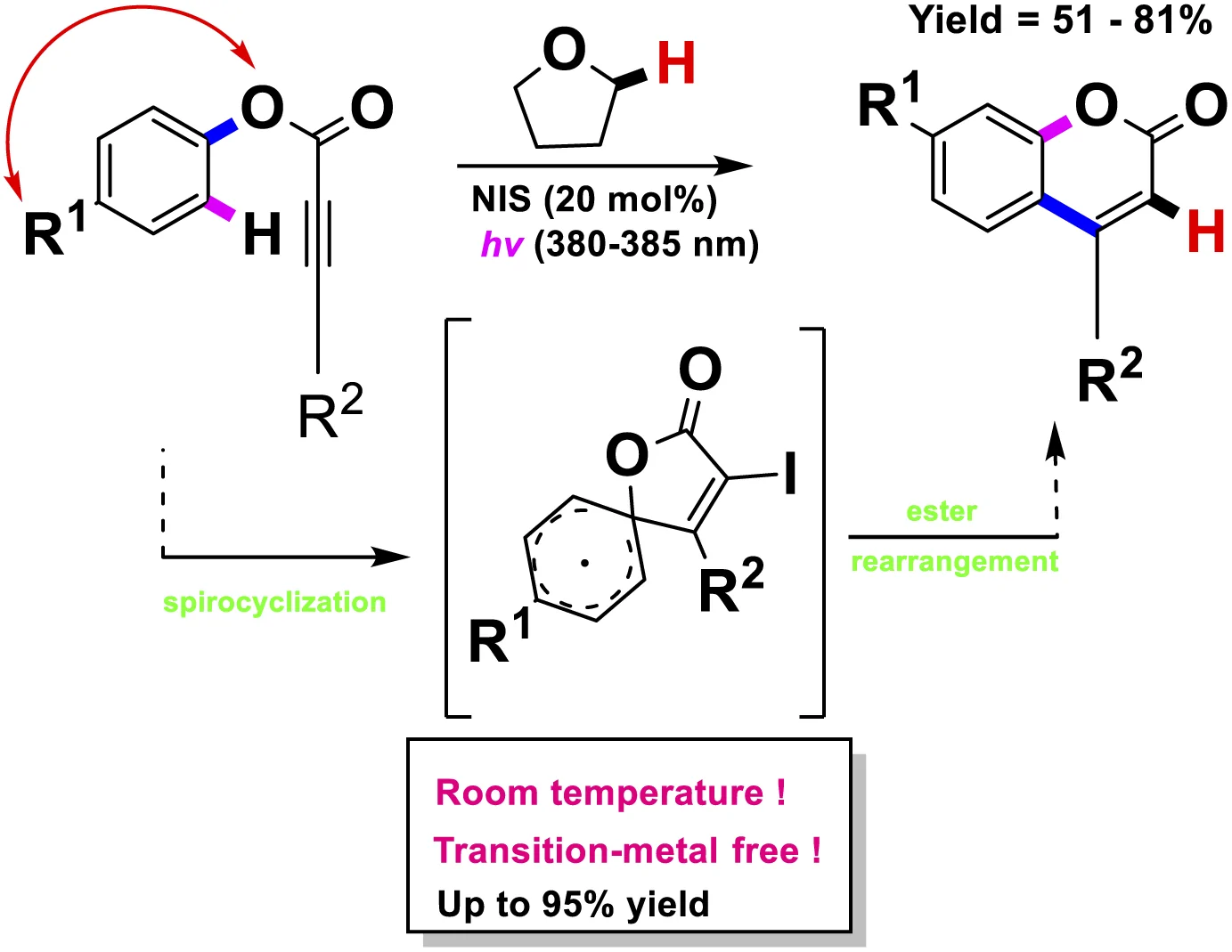
Synthesis of coumarin from the photoinduced cyclization of alkynoates

### 6.5. Synthesis of 3-imidazolyl coumarin under solvent-free conditions

Zhou and his team developed a 3,7-disubstituted coumarin under solvent-free conditions, achieving high quantum yields.^47^ The optical aspects of these fluorescent compounds were investigated, revealing an uncommon display of solution and solid dual efficient luminescence. In organic solvents, the fluorescent molecule 3-imidazolyl coumarins displayed blue fluorescence at 470 nm and yellow luminescence in solid form. The fluorescence quantum yields increased up to 0.9 by adding a diethylamino group to the coumarins' 7-position (Scheme 11). Furthermore, all structural optimizations were performed using time-dependent density functional theory (TD-DFT), and the resulting optical properties matched the experimental results. According to the article, leveraging the intramolecular charge transfer (ICT) effect offers an efficient approach to fabricating dual-state, high-luminescent materials, which are highly useful precursors for producing Organic Light-Emitting Diodes and fluorescent probes.^48^

**Scheme 11:**
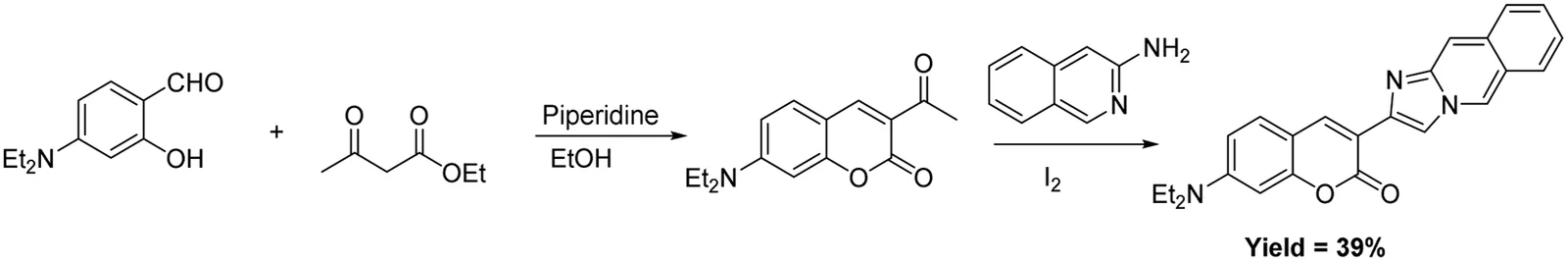
The synthesis of 2-acetylcoumarin and 3-imidazolyl coumarin.

### 6.6. Synthesis using Michael alkylation reaction

Several unique techniques have been reported after Widman's pioneering work, which employed the reaction of 3-acylcoumarins with α-haloketones to generate tricyclic coumarins.^49^ Coumarins with an electron-withdrawing group (-CN) at position 3 WG were made to react with brominated β-diketone under Michael alkylated conditions to provide an appealing pathway to tricyclic coumarins. Based on the stated assumption, Sun and his co-workers began their research with 3-cyanocoumarin as the model substrate and obtained exceptional results when they reacted it with 2-bromomalonate (Scheme 12).^50^

**Scheme 12:**
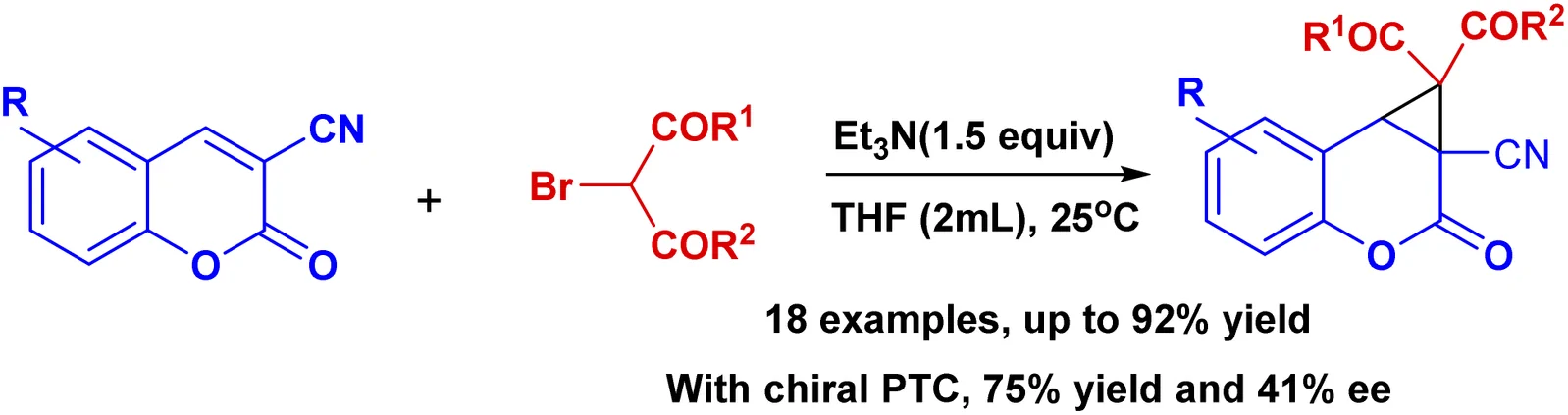
Cyclopropanation of 3-substituted coumarins.

### 6.7. *p*-Toluene sulfonic acid catalytic synthetic approach

In this study by Winata and team, *p*-toluene sulfonic acid (PTSA) was used in catalytic amounts in an 800 W microwave-assisted reaction to successfully synthesize 4-methyl umbelliferone. Spectroscopic techniques were used to further interpret the synthesized chemical.^51^ Resorcinol and ethyl acetoacetate had been successfully combined as a precursor to create 4-methyl umbelliferone; the product was obtained in 48.36% yield, which was lower than that obtained when InCl_3_ was used as a Lewis acid catalyst.^52^ (Scheme 13). Based on product yield, this finding demonstrated that the Brønsted acid catalyst is less reactive than the Lewis acid catalyst during coumarin synthesis. Moreover, this substance showed little effect against the bacteria that cause acne, which was the aim of this work.

**Scheme 13:**
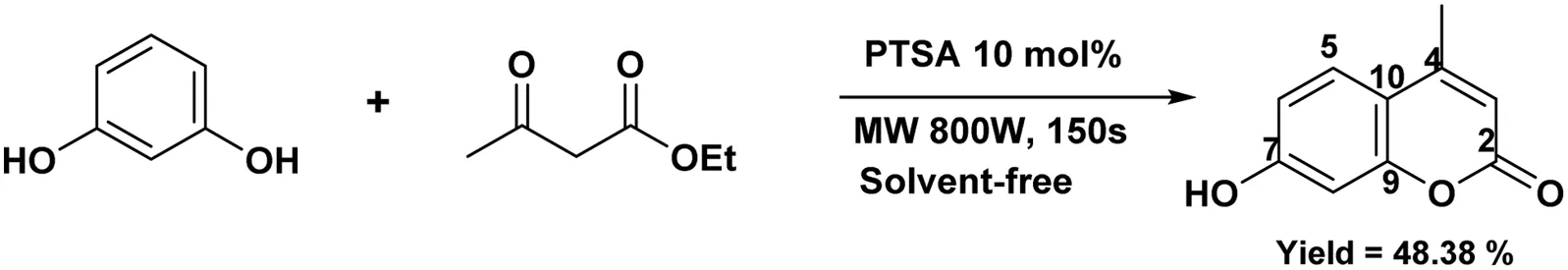
Formation of 7-hydroxy-4-methylcoumarin compound.

### 6.8. Condensation reaction of coumarin-3-carboxylic acids

Recent studies have reported the use of water extract of rice straw (WERS) as a base catalyst in several base-catalyzed reactions. A sustainable method for producing coumarin-3-carboxylic acids in clean rice straw husk water extract was reported in an article by Patil and co-authors (Scheme 14). The authors demonstrate that this approach afforded coumarin-3-carboxylic acids in 72–94% isolated yields.^53^ This approach is advantageous and appealing, with benefits for the economy and the environment. It is an easy and environmentally friendly approach to creating the desired compounds.

**Scheme 14:**
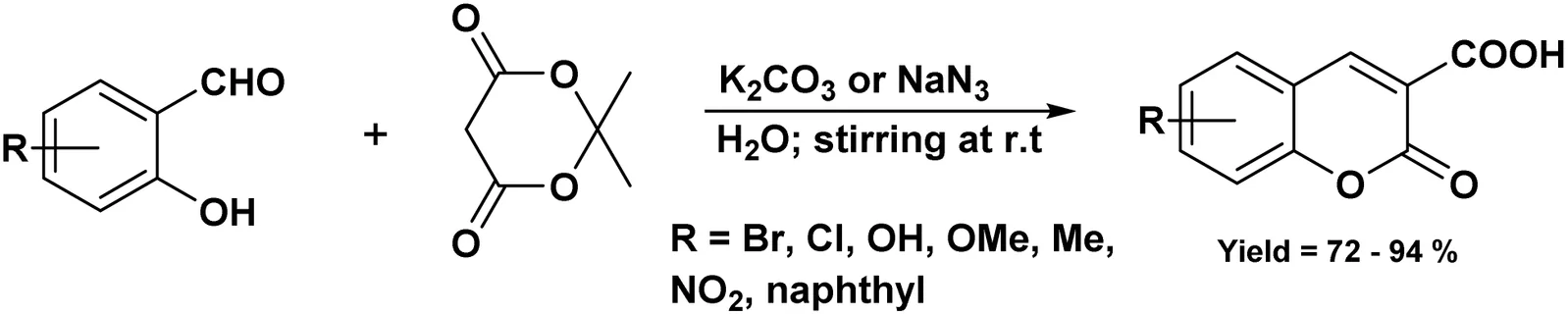
Water-based one-pot synthesis of coumarin-3-carboxylic acids.

### 6.9. Condensation-based benzyl pyrazolyl coumarin synthesis

In an earlier attempt to develop an improved bioactive scaffold, Ghosh and co-workers reported the synthesis of benzylpyrazolyl coumarins by combining a pyrazolone with a 3-benzyl-substituted coumarin. Glacial acetic acid was used as a catalyst, and the synthesis was carried out in an aqueous medium.^54^ The use of other catalysts like ZnO and ZrO_2_ for the synthesis of benzyl pyrazolyl was reported, but due to Amberlite IR-120 resin's low cost, reusability, the convenience of use, and ease in isolation upon reaction completion, it has become a significant heterogeneous catalyst in organic synthesis. Using Amberlite IR-120 as a catalyst, Katariya and his colleagues demonstrated an alternative protocol for a sustainable, eco-friendly, and relatively inexpensive synthetic route to benzyl pyrazolyl coumarin in good yield. It involved a multi-component reaction that exhibited significant benefits, such as shorter reaction time, simpler operation, and the low cost of the catalyst and aqueous medium (Scheme 15).^55^

**Scheme 15:**
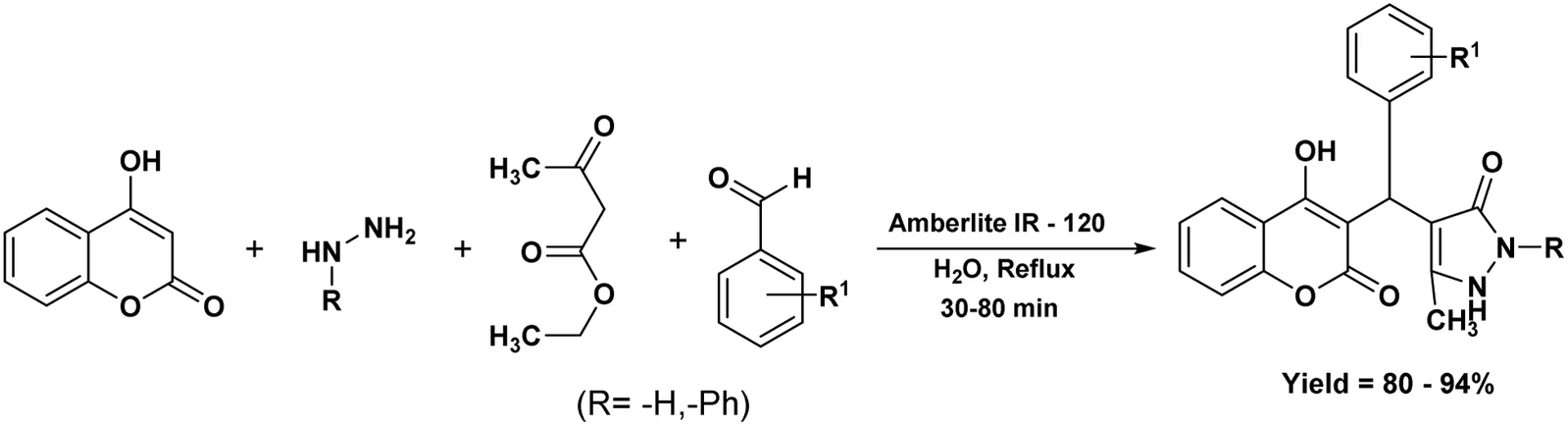
Synthesis of benzylpyrazolyl coumarin.

### 6.10. Cyclization using diaryl diselenides on an aryl ether

Radical cyclization under mild conditions has emerged as an effective approach for the synthesis of cyclic compounds, including 3-substituted coumarins. Given their ongoing research in the preparation of sulphur- or selenium-based products, Fang and co-authors presented a highly effective approach for producing 3-organoselenyl-2*H*-coumarins *via* radical cyclization of propargylic aryl ether with diaryl diselenides, together with the concomitant production of C=O double bonds under metal-free conditions (Scheme 16).^56^ Excellent functional group compatibility and the formation of many bioactive substrates in excellent yields indicate that this transformation may be used in medicinally relevant and biologically active compounds for drug design.

**Scheme 16:**
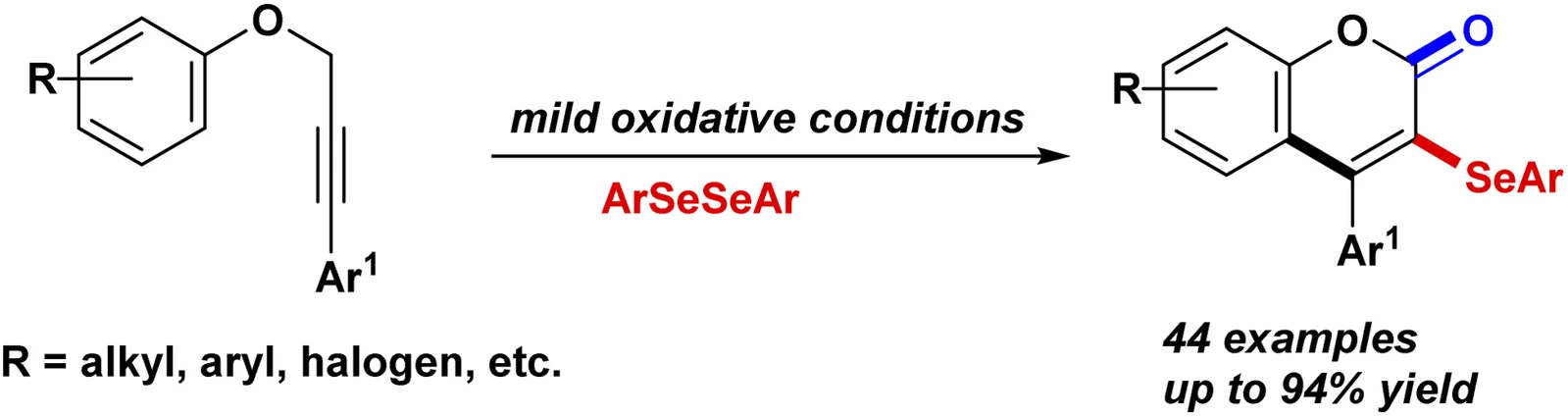
3-Selenylated coumarin synthesis.

### 6.11. Cyclization of aryl 3-arylpropynoates into 4-arylcoumarins

There have not been many Pt(ii) complexes created for organic synthesis catalysis. Nevertheless, since the discovery of its anticancer potential, various Pt(ii) compounds have been developed as substitutes for the widely used anticancer drug cisplatin. As a result, Zaitceva investigated Pt(ii) derivatives on the theory that a particular ligand may be used to tailor Pt(ii) reactivities and solubility. It was shown that cyclometalated platinum(ii) complexes exhibited increased reactivity, yielding a variety of coumarins in good to high yields (Scheme 17). The 2-(pyridin-2-yl) phenylPt(ii)(acetonitrile) chloride compound was the most practical and productive of the Pt(ii) catalysts that were tested. Significant yields of benzocoumarins could be obtained using this readily available compound, and coumarins were recovered with yields up to 94%. Thus, this platinum catalyst outperforms other recognized catalysts, such as the simple platinum chloride catalysts, in terms of competitiveness.^57^ In this study, monomeric or dimeric platinacycles were used as a catalyst to create effective and gentle access to coumarins. Various substituents and functional groups demonstrated the ability to cyclize, yielding a wide range of substituted coumarins. By using the same platinum-catalyzed method, very high yields of benzocoumarins could also be obtained.

**Scheme 17:**
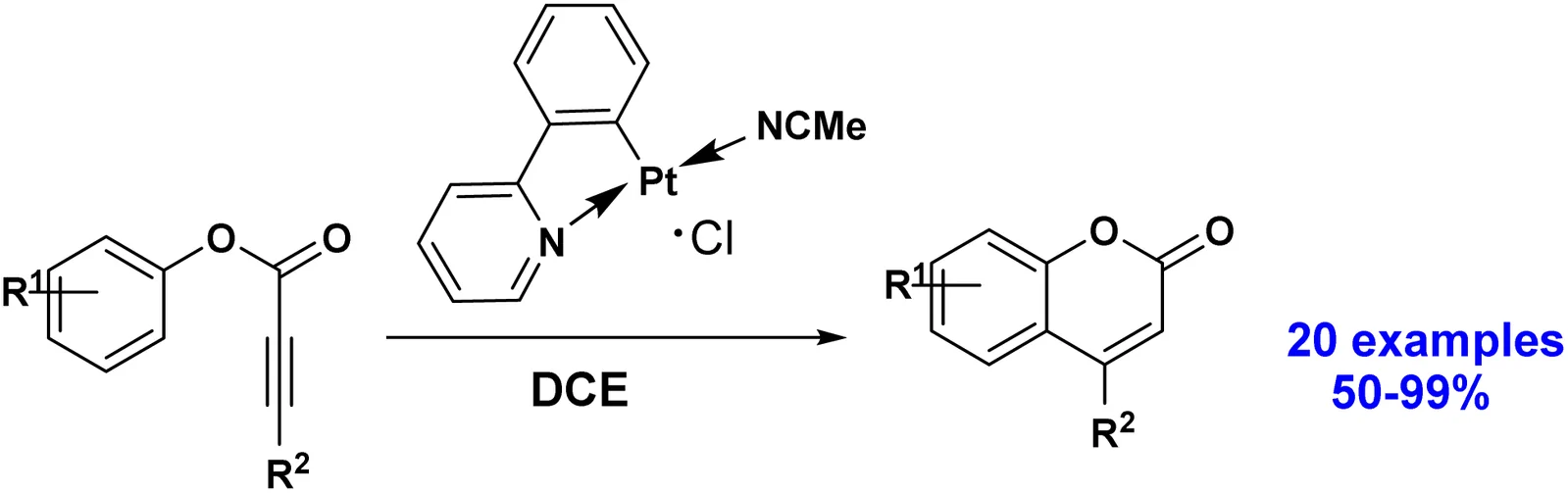
Aryl propynoate cyclizations induced by metals.

### 6.12. Synthesis *via* monofluoromethylation of alkynoates with a silver catalyst

Fu, Sun and Li subjected Alkynoates to a cascade radical monofluoromethylation reaction with sodium monofluoromethanesulfinate (CH_2_FSO_2_Na) that was catalyzed by silver. This reaction produced a range of 3-monofluoromethylated coumarins. Potassium persulfate, K_2_S_2_O_8_, and a silver catalyst both worked well to speed up the reaction (Scheme 18).^58^ This approach also has a simple operation, mild reaction conditions, and excellent functional group tolerance.

**Scheme 18:**
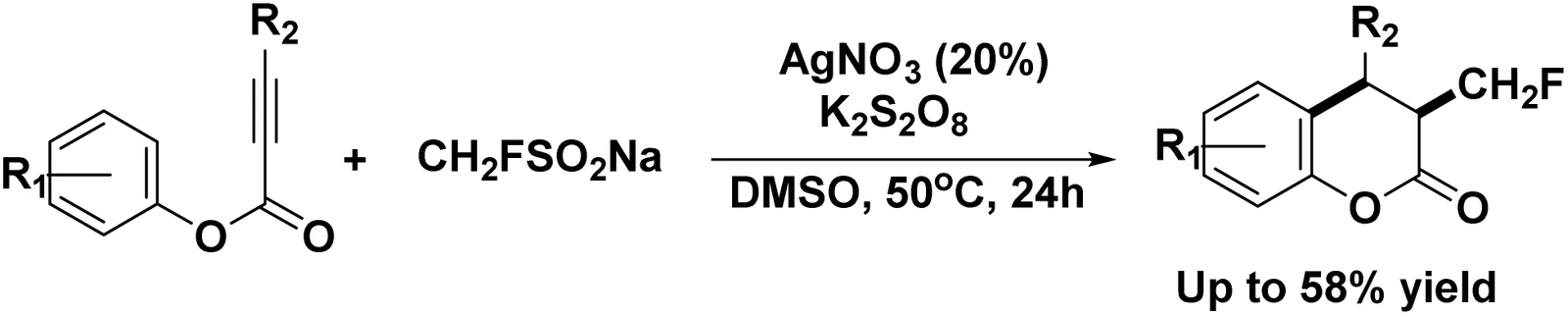
The synthetic reaction using a silver catalyst.

### 6.13. Synthesis using a photocatalyst-free visible light approach

Without the need for a photocatalyst, Xi and his colleagues described a moderate and effective visible-light-enhanced preparation of C-3 dicarbon coumarins from 3-arylacetylene coumarins.^59^ 3-Arylacetylene coumarins, I_2_, NaHCO_3_, and DCE: H_2_O were put into a reaction tube with a magnetic stirring bar, and they were swirled under a 3 W blue LED in an oxygen atmosphere and at ambient condition in one day. The mixture was dried under vacuum, and effective purification of the crude residue was achieved with flash chromatography (Scheme 19).

**Scheme 19:**
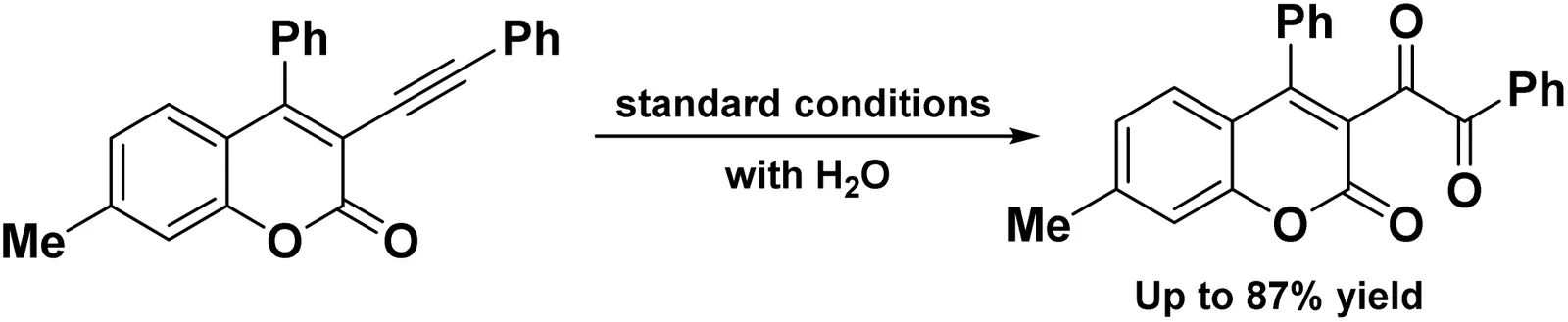
Synthesis of C-3 dicarbonyl coumarins.

### 6.14. Synthesis using the microwave-assisted method under different conditions

Given the significance of furocoumarins, Kaneria and team's ongoing interest in constructing heterocyclic compounds based on coumarins prompted them to focus their efforts on developing an effective approach to access furan-fused coumarins (Scheme 20). Under microwave irradiation, two methodologies are used. The Nef reaction condition and the Feist–Benary reaction condition have been used to synthesize the target compounds.^60^ Two different methods were used for the synthesis by varying the reaction catalysts. Method A used piperidine while method B used CH_3_COOH in CH_3_COONH_4_ in Feist–Benary condition.

**Scheme 20:**
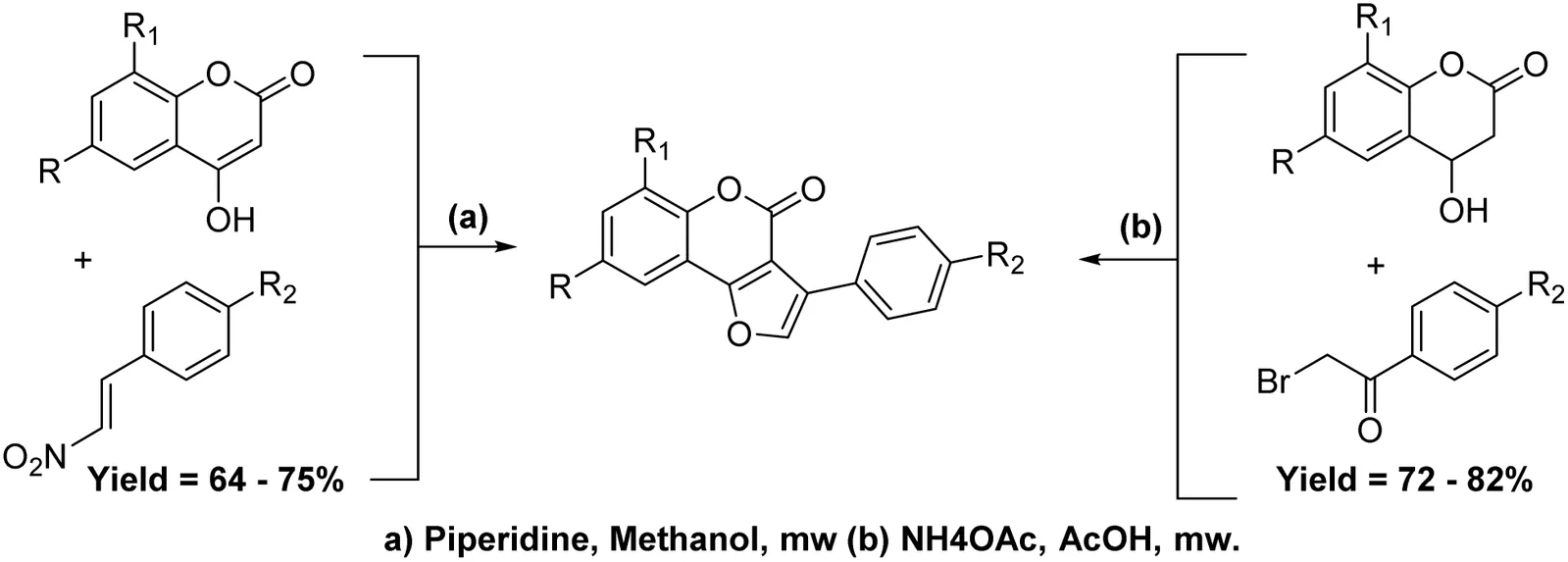
Synthesis of 3-aryl-furo[3,2-*c*] coumarins.

### 6.15. Coumarin synthesis catalyzed by lipase TL IM

The enzymatic synthesis of coumarin carboxamide derivatives in two steps was demonstrated by Du and his team, using *Thermomyces lanuginosus* lipase TL IM to catalyze the reaction between salicylaldehyde and dimethyl malonate to produce a coumarin carboxylate methyl intermediate, which was subsequently transformed into carboxamide scaffolds (Scheme 21).^61^ A significant new process is produced by coupling enzymes with continuous-flow microreactors, as used in biocatalysis to boost overall reaction efficiency, make biocatalyst recycling easier (without mechanical stirring), and streamline the reaction procedure. In addition, using enzymes as catalysts reduces environmental contamination and avoids laborious reaction procedures and challenging follow-up processing. Under optimized continuous-flow conditions, the coumarin carboxylate methyl intermediates were obtained in 82.6–89.3% isolated yields, depending on the salicylaldehyde substrate. For the subsequent enzymatic amidation, the optimized continuous-flow process afforded the model isobutyl coumarin-3-carboxamide in 85.7 ± 0.6% isolated yield, compared with 66.9 ± 1.2% in the conventional shaker reactor. Across the substrate scope, the final coumarin carboxamides were obtained in yields ranging from <5% to 90.5%.^62^ This work provides knowledge that makes it easier to design and synthesize new coumarin compounds for the next round of drug screening.

**Scheme 21:**
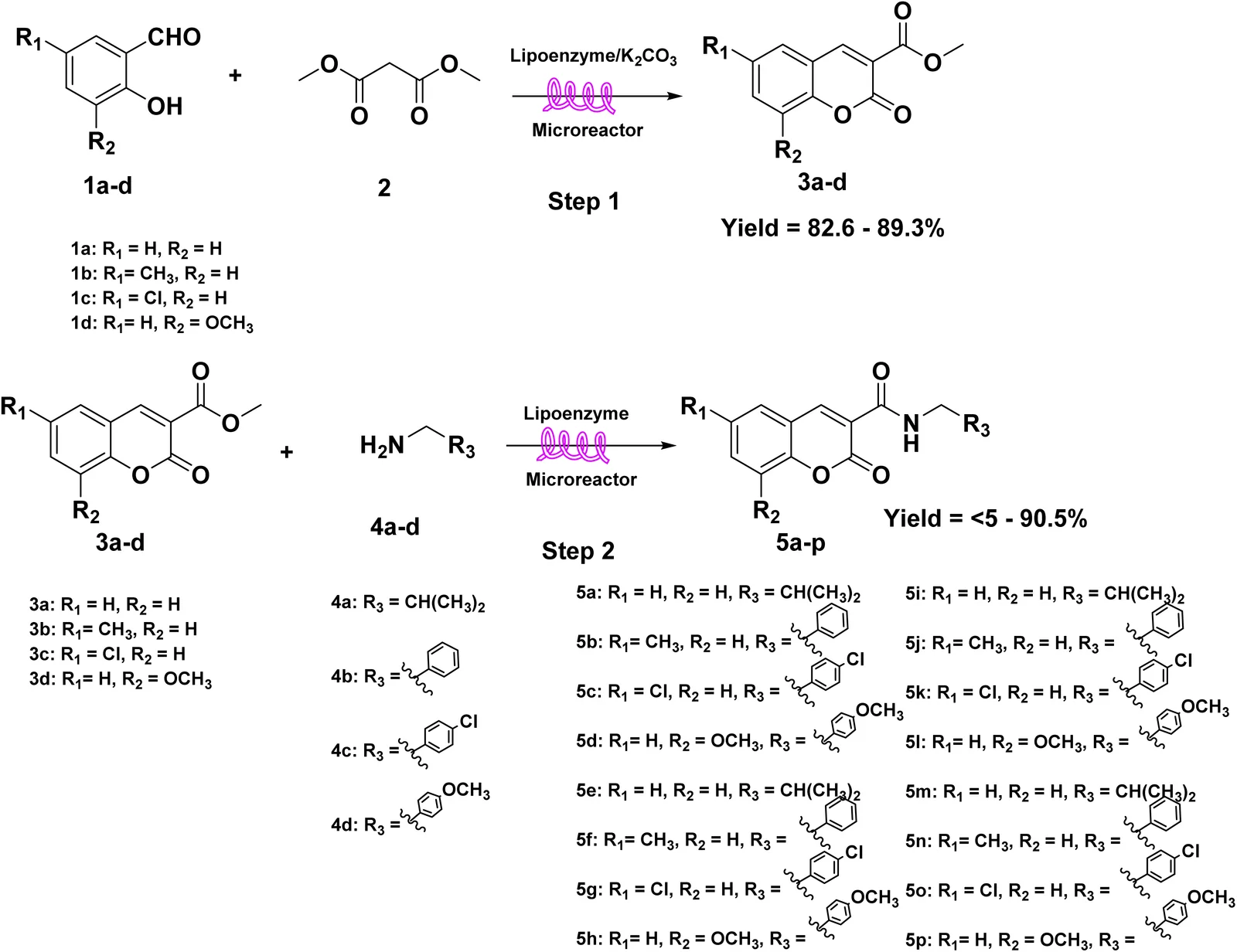
Synthesis of coumarin carboxamide derivatives.

## 7. Pharmacological and therapeutic properties of coumarin motifs

The structural versatility of the coumarin motif, together with its amenability to substitution and hybridization, has driven extensive exploration of its therapeutic potential beyond anticoagulation. Recent studies have investigated coumarin-based compounds across a broad range of therapeutic areas, including infectious, inflammatory, neurological, metabolic, and malignant disease (Table 1). Accordingly, this section considers the progression from established coumarin-derived medicines with clinical usage to recent experimental leads. This progression also provides insight into how structural modification of the coumarin nucleus influences pharmacological activity and supports the development of new therapeutic applications.

**Table 1:** Summary of pharmacological activity of clinically established coumarins and investigational leads

Coumarin/derivative	Source	Disease/disorder/activity	Experimental model	Quantitative activity	Mechanism/target/relevant SAR	Toxicity/safety	Reference
Warfarin (2)	Synthetic	Anticoagulant; thromboembolic disorders	Clinical use	*t*½ = 20–60 h	4-Hydroxycoumarin core; C3 1-phenyl-3-oxobutyl group; VKORC1 inhibition	Bleeding risk; narrow therapeutic window; drug and dietary interactions	63 and 64
Acenocoumarol (3)	Synthetic	Anticoagulant; thromboembolic disorders	Clinical use	*t*½ = 8–11 h	4-Hydroxycoumarin core; C3 1-phenyl-3-oxobutyl + *p*-NO_2_ phenyl; VKORC1 inhibition	Bleeding risk; narrow therapeutic window; drug and dietary interactions	63 and 64
Phenprocoumon (4)	Synthetic	Anticoagulant; thromboembolic disorders	Clinical use	*t*½ ≈ 5 days	4-Hydroxycoumarin core; C3 1-phenylpropyl; VKORC1 inhibition	Bleeding risk; narrow therapeutic window; drug and dietary interactions	63 and 64
Novobiocin (5)	Clinical aminocoumarin antibiotic	Bacterial infection	Clinical use against susceptible *S. aureus*	—	Inhibits ATP-binding site of bacterial DNA gyrase B	Hepatic and haematological toxicity; resistance; US approval withdrawn	65
Methoxsalen (8-methoxypsoralen) (6)	Furanocoumarin	Psoriasis, vitiligo and skin manifestations of cutaneous T-cell lymphoma	UVA photochemotherapy	—	Photoactivation produces DNA photoadducts, inhibiting DNA synthesis and cell proliferation	—	66
7a	Synthetic	Alkaline phosphatase inhibition	h-TNAP	IC_50_ = 0.38 ± 0.01 µM	Methylbenzenesulphonate substituent; sulphonate identity influenced activity	—	67
7b	Synthetic	Alkaline phosphatase inhibition	h-IAP	IC_50_ = 0.45 ± 0.02 µM	4-Fluorobenzenesulphonate substituent; isozyme selectivity reported	—	67
8	Synthetic	Antimalarial	*Plasmodium falciparum* 3D7	IC_50_ = 1.7 µM	Acyclic α-aminohydroxycoumarin; possible hemozoin inhibition and DNA interaction; intramolecular N–H⋯O–H H-bond proposed to contribute	—	68
9	Synthetic	Antibacterial	*E. coli*, *S. aureus*	MIC = 32 µg mL^−1^ (*E. coli*); 128 µg mL^−1^ (*S. aureus*)	Coumarin-isoxazole-sulfonamide; *p*-methylphenyl substitution associated with strong activity	—	69
10	Synthetic	Anti-*H. pylori*/urease-associated antibacterial activity	*Helicobacter pylori*	Strong antibacterial activity reported; specific quantitative value not reported in current write-up	Carboxylic acid identified as critical for *anti-H. pylori* activity; urease inhibition proposed to contribute to antibacterial effect	—	70
11 and 12	Synthetic/investigated coumarins	Carbonic anhydrase inhibition; potential antibacterial relevance	Carbonic anhydrase inhibition studies	Specific quantitative values not reported in current write-up	Prodrug inhibitors; CA-mediated lactone hydrolysis generates 2-hydroxycinnamic acids that bind at the CA active-site entrance; variable entrance region contributes to isoform selectivity	—	71
13	Synthetic	Bacterial carbonic anhydrase inhibition/potential antibacterial activity	*Neisseria gonorrhoeae* NgCAα	*K*_i_ = 28.6 µM; most potent of 14 tested	4-Methyl and 7-diethylamino substitution; bacterial CA inhibition; preferential activity over human CA I and II	—	71 and 72
14	Synthetic	Antibacterial	*S. aureus, MRSA, and E. coli*	*S. aureus*: MIC = 4.88 µg mL^−1^, MBC = 9.76 µg mL^−1^; MRSA: MIC = 39.06 µg mL^−1^, MBC = 76.7 µg mL^−1^; *E. coli*: MIC = 78.13 µg mL^−1^	Coumarin-based heterocyclic derivative; stronger activity against the tested Gram-positive bacteria	—	73
15	Synthetic	Antifungal	*Fusarium moniliforme*	74% inhibition at 0.5 mg mL^−1^	Coumarin-thiazole derivative; CF_3_ group associated with enhanced activity	—	74
16	Synthetic	Antifungal	*F. graminearum*, *Curvularia lunata*	89% and 93.4% inhibition, respectively, at 0.5 mg mL^−1^	Coumarin-thiazole derivative; CF_3_ substitution enhanced activity	—	74
17	Synthetic	Antitubercular	*Mycobacterium tuberculosis* H37Rv	MIC = 0.8 µg mL; streptomycin = 3.125 µg mL^−1^	Coumarinyl pyrazoline bearing aminobenzoic acid; aminobenzoic acid improved activity	—	75
18	Synthetic	Antitubercular	*M. tuberculosis H37Rv*; ΔkatG *mutant*; *infected macrophages*; *InhA assay*	H37Rv MIC = 15 µg mL^−1^; InhA IC_50_ = 0.565 µM	InhA inhibition; tetrazole-containing 4-phenylcoumarin derivative	4-Phenylcoumarin derivativeIntracellular macrophage evaluation reported; further safety characterization require	76
19a	Synthetic	Antileishmanial	*Leishmania panamensis* amastigotes	EC_50_ = 2.1 ± 0.1 µM	3-Hydroxyphenyl coumarin-chalcone	—	77
19b	Synthetic	Antileishmanial	*L. panamensis* amastigotes	EC_50_ = 2.5 ± 0.2 µM	3-Methoxyphenyl coumarin-chalcone	—	77
19c	Synthetic	Antileishmanial	*L. panamensis* amastigotes	EC_50_ = 3.7 ± 0.5 µM	Unsubstituted phenyl coumarin-chalcone	—	77
20a	Synthetic	Antileishmanial	*L. major promastigotes and intracellular amastigotes*	IC_50_ = 1.21 ± 0.18 µM (promastigote), 2.41 ± 0.22 µM (amastigote)	Antifolate mechanism; PTR1/DHFR-TS implicated; *N*-unsubstituted 5-nitroisatin derivatives bearing p-F/p-Cl favoured activity	—	78
20b	Synthetic	Antileishmanial	*L. major promastigotes and intracellular amastigotes*	IC_50_ = 1.43 ± 0.26 (promastigote),and 3.78 ± 0.28 µM (amastigote)	Antifolate mechanism; PTR1/DHFR-TS implicated; *N*-unsubstituted 5-nitroisatin derivatives bearing *p*-F/*p*-Cl favoured activity	—	78
Bichromonol (21)	Natural; isolated from stem bark of *Hypericum roeperianum*	Anti-HIV-1	Infected MT-4 cells	EC_50_ = 8.7 µM against HIV-1IIIB; 6.6–12.0 µM against resistant variants	Dimeric coumarin; activity against HIV–1IIIB and resistant variants	CC_50_ = 54 µM in MT-4 cells	79
22a	Synthetic	Antiviral	coronavirusHCoV-OC43; SARS-CoV-2-infected cells; additional coronavirus modelsHCoV-OC43	IC_50_ = 39.8 µg mL^−1^	Synthetic coumarin analogue; inhibition of coronavirus replication investigated using plaque/qRT-PCR and cellular assays	HEK-293 CC_50_ = 500.55 µg mL^−1^	80
22b	Synthetic	Antiviral	coronavirusHCoV-OC43; SARS-CoV-2-infected cells	IC_50_ = 41 µg mL^−1^	Synthetic coumarin analogue; inhibition of coronavirus replication investigated using plaque/qRT-PCR and cellular assays	—	80
23	Synthetic	Antiviral; *anti*-TMV	Tobacco mosaic virus plant model	Therapeutic EC_50_ = 180.7 µg mL^−1^; protective EC_50_ = 190.3 µg mL^−1^	Coumarin heterocyclic derivative; activity demonstrated in a plant-virus model	Therapeutic relevance to mammalian systems not established	81
24	Synthetic organoselenium coumarin	Chemotherapy adjuvant/myeloprotection	Female Swiss albino mice; MCF-7, Colo-205 and Dalton's lymphoma	6 mg kg^−1^ MUS with 12 mg kg^−1^ carboplatin	Redox modulation; suppression of ROS/RNS and restoration of glutathione balance; enhanced carboplatin activity	Myeloprotective; reduced chromosomal aberrations, micronucleus formation and DNA damage; restored bone-marrow cellularity	82
25	Synthetic	Anticancer; NSCLC	FAK assay; H1299 NSCLC cells; mouse tumour model	FAK IC_50_ = 4.968 nM; H1299 IC_50_ = 0.28 µM	FAK inhibition; induced apoptosis/cell-cycle arrest and inhibited migration/invasion	*In vivo* tolerability evaluated in tumour-bearing mice	83
26	Synthetic	Antiproliferative/anticancer research	NCI-60 panel	—	Coumarin-annulated stilbene; proposed relevance to oxysterol-binding proteins and *trans*-Golgi trafficking; fluorescent mechanistic probe	—	84
27	Synthetic	Anti-inflammatory	Carrageenan-induced paw oedema in rats	71.94–77.96% inhibition at 200 mg kg^−1^; greater than celecoxib at reported time point	COX-related activity supported by docking; NO_2_ and Cl substitution favoured activity	—	85
28	Synthetic	Analgesic/anti-inflammatory	5-LOX inhibition and ulcerogenic assessment	5-LOX IC_50_ = 2.09 nM	Coumarin-benzothiazole; C6–Cl or –OCH_3_ substitution favoured 5-LOX inhibition	Fewer gastric lesions than acetylsalicylic acid	86
29	Synthetic	Antidepressant	Forced swim and tail suspension tests in mice	DID = 26.09 at 40 mg kg^−1^; fluoxetine DID = 28.80	4-Fluorobenzyl-piperazine substitution at C7; proposed 5-HT1A receptor engagement; increased brain 5-HT	Favourable BBB permeability reported; specific toxicity not reported	47
30	Synthetic	Potential anti-Alzheimer's	AChE inhibition assay	AChE IC_50_ = 29.56 µM; SI = 1.552	Coumarin-1,3,4-oxadiazole hybrid; docking −10.1 kcal mol^−1^	—	87
31	Synthetic	Potential anti-Alzheimer's	BuChE inhibition assay	BuChE IC_50_ = 23.97 µM	Coumarin-1,3,4-oxadiazole hybrid; multi-target-directed design	—	87
32	Synthetic	Inflammation-associated depression	IDO1/TDO assays; LPS-stimulated BV2 cells; LPS-induced mouse modelIDO1	IC_50_ = 0.34 µM; TDO IC_50_ = 0.75 µM; active at 20 mg kg^−1^ in mice	Dual IDO1/TDO inhibition; modulation of kynurenine/serotonin and BDNF/PKA signalling	Experimental PK and mouse evaluation reported	88
33 and 34	Natural/derived compounds	Antidiabetic	Reviewed enzyme/mechanistic studies	—	α-Glucosidase inhibition; coumarin, umbelliferone and coumarin-3-carboxylic acids associated with suppression of gluconeogenesis	—	89
35	Synthetic	Antidiabetic/α-amylase inhibition	α-Amylase inhibition assay (DNSA method)	IC_50_ = 67.52 µM; metformin = 54.13 µM	3,5-Dimethoxyphenyl carboxamide; docking supported α-amylase binding	—	90
36	Synthetic	Antidiabetic	α-Amylase and α-glucosidase enzyme assays	(a): IC_50_ = 0.70 ± 0.05 µM (α-amylase), 1.10 ± 0.10 µM (α-glucosidase); (b): 0.90 ± 0.05 and 1.20 ± 0.10 µM	Dual α-amylase/α-glucosidase inhibition; p-CF_3_ in 1 and 3-OH/4-F in 16 associated with high activity	Not established in reported biological evaluation	91
37c	Synthetic	Antioxidant	DPPH assay	IC_50_ = 3.87 µg mL^−1^; ascorbic acid = 2.83 µg mL^−1^	6-Cl coumarin + 4-hydroxyphenyl sulfonamide	—	16
37d	Synthetic	Antioxidant	DPPH assay	IC_50_ = 4.30 µg mL^−1^; ascorbic acid = 2.83 µg mL^−1^	6-Me coumarin + 4-hydroxyphenyl sulfonamide	—	16
Suberosin (38)	Natural; isolated from aerial parts of *Ferulago carduchorum*	Anticoagulant	Rats; prothrombin time	Significant PT prolongation at 3 and 6 mg kg^−1^; 17.4 s at 6 mg kg^−1^	Natural coumarin; anticoagulant activity assessed by PT	Specific toxicity of isolated suberosin not reported; parent extract LD_50_ > 2000 mg kg^−1^	15
Suberenol (39)	Natural; isolated from aerial parts of *F. carduchorum*	Anticoagulant	Rats; prothrombin time	Significant PT prolongation at 3 and 6 mg kg^−1^	Natural coumarin; anticoagulant activity assessed by PT	Specific toxicity of isolated suberenol not reported; parent extract LD_50_ > 2000 mg kg^−1^	15

### 7.1. Clinically established coumarin-derived drugs

Coumarins have a long history of medicinal application, most notably through the 4-hydroxycoumarin anticoagulants. Their development arose from investigations of haemorrhagic disease in cattle associated with spoiled sweet-clover hay. In 1939, Link and colleagues isolated dicoumarol 1 as the anticoagulant responsible for this effect, and subsequent synthesis of related 4-hydroxycoumarins led to the identification of warfarin. Initially introduced as a rodenticide in 1948, warfarin subsequently entered clinical use and was approved for human use in the United States in 1954.^92^

Warfarin 2, phenprocoumon 3 and acenocoumarol 4 (Fig. 2) share the 4-hydroxycoumarin pharmacophore and inhibit vitamin K epoxide reductase complex subunit 1 (VKORC1), reducing regeneration of reduced vitamin K required for γ-carboxylation of coagulation factors II, VII, IX, and X and proteins C and S.^63,64^ The established SAR of this series indicates that the 4-hydroxycoumarin core is important for vitamin K antagonism, whereas modification of the C3 substituent influences target inhibition and pharmacokinetic behaviour.^64^ Warfarin bears a 1-phenyl-3-oxobutyl substituent at C3 and contains a chiral centre, with S-warfarin being more potent than R-warfarin. Acenocoumarol has the same 1-phenyl-3-oxobutyl C3 side chain as warfarin but incorporates a para-nitro group on the phenyl ring and has a shorter elimination half-life of 8–11 h, compared with an effective half-life of 20–60 h for warfarin. Phenprocoumon instead bears a 1-phenylpropyl substituent at C3 and has a considerably longer elimination half-life of approximately 5 days. These agents are used for the prevention and treatment of thromboembolic disorders, but their clinical application is limited by narrow therapeutic windows, drug and dietary interactions, and bleeding risk.^63,64^

**Fig. 2:**
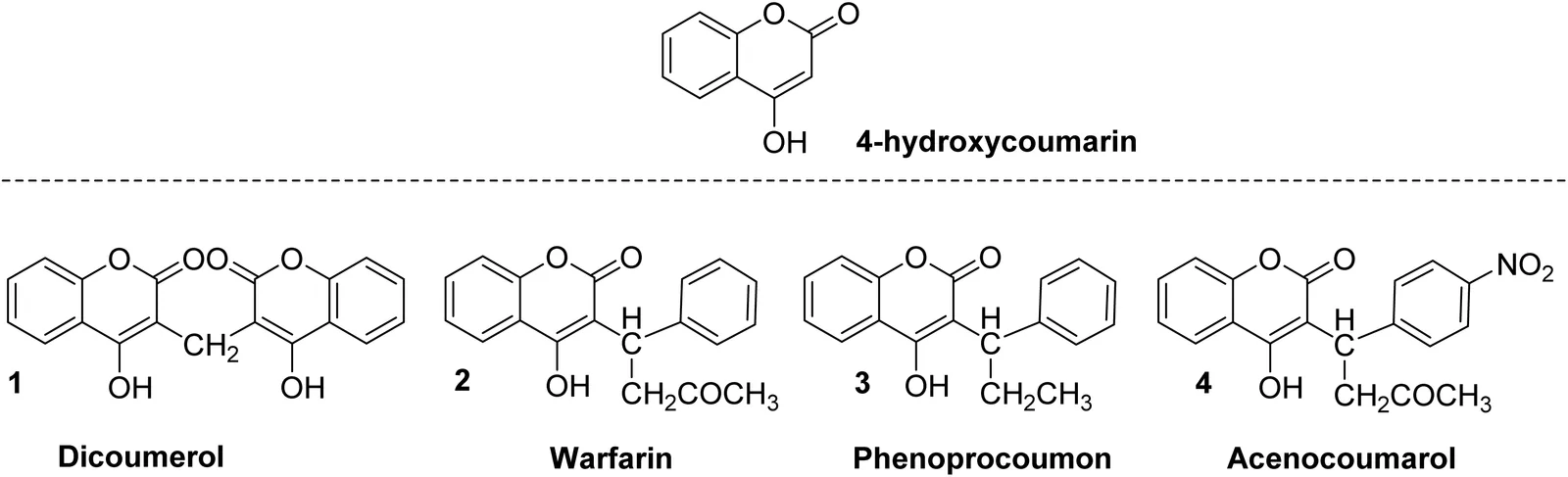
Chemical structures of 4-hydroxycoumarin anticoagulants.

Aminocoumarin 5 and furanocoumarin 6 (Fig. 3) derivatives have also produced clinically relevant therapeutics. Novobiocin, an aminocoumarin antibiotic, was used against susceptible *Staphylococcus aureus* infections by inhibiting the ATP-binding site of bacterial DNA gyrase B. Its clinical use was limited by adverse effects, including hepatic and haematological toxicity, and the development of bacterial resistance, and its US approval was subsequently withdrawn.^65^ Methoxsalen (8-methoxypsoralen) has a distinct application as a furanocoumarin photochemotherapeutic. It is used with UVA irradiation for severe psoriasis, vitiligo, and the skin manifestations of cutaneous T-cell lymphoma, with photoactivation producing DNA photoadducts that inhibit DNA synthesis and cell proliferation.^66^

**Fig. 3:**
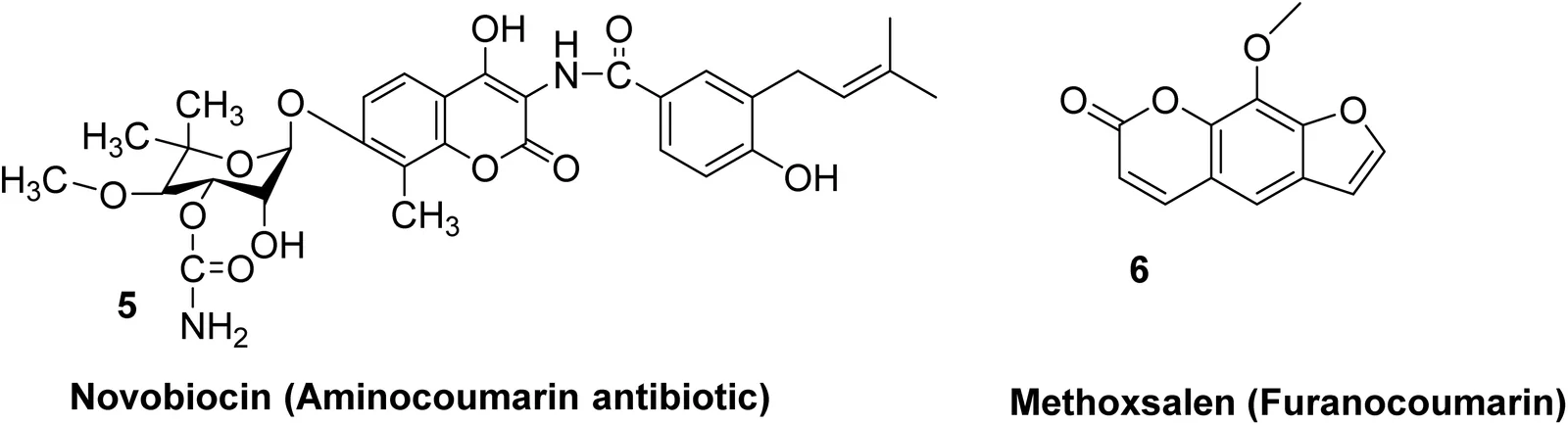
Clinically established aminocoumarin and furanocoumarin derivatives.

### 7.2. Other therapeutic activities of investigated coumarin derivatives

The structural versatility of the coumarin scaffold has continued to drive investigation across diverse therapeutic areas, yielding numerous leads and provide a basis for continued development.

Coumarin derivatives have been investigated against several therapeutically relevant enzymes, with inhibitory activity strongly influenced by substitution pattern and scaffold architecture. Iqbal *et al.* evaluated tricyclic coumarin sulphonates against human tissue-nonspecific alkaline phosphatase (h-TNAP) and human intestinal alkaline phosphatase (h-IAP) (Fig. 4). The methylbenzenesulphonate 7a was the most potent h-TNAP inhibitor (IC_50_ = 0.38 ± 0.01 µM), whereas the 4-fluorobenzenesulphonate 7b was most active against h-IAP (IC_50_ = 0.45 ± 0.02 µM). Some derivatives also showed isozyme selectivity, with SAR and docking analyses indicating that sulphonate substituent identity contributed substantially to inhibitory activity. These finding highlight the potential of coumarin derivatives to modulate alkaline phosphatase activity and identify this enzyme class as an additional pharmacologically relevant target for further investigation.^67^

**Fig. 4:**
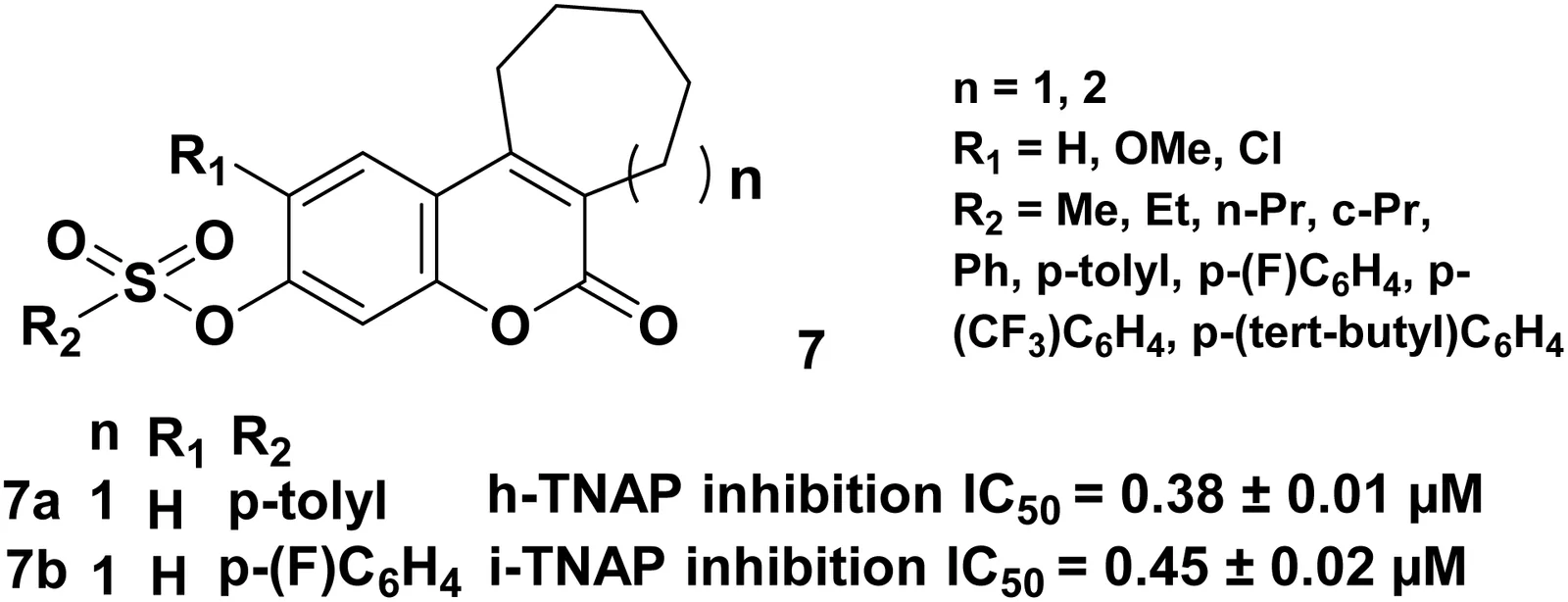
Tricyclic coumarin sulphonates derivatives with alkaline phosphatase enzyme inhibitory activity.

### 7.3 Antimalarial activities

Mbaba and colleagues synthesized coumarin-based ferrocenyl 1,3-oxazine hybrids and evaluated their activity against *Plasmodium falciparum* 3D7. Compound 8 was the most active derivative in the α-aminohydroxycoumarin series, with an IC_50_ of 1.7 µM. SAR analysis showed that the acyclic α-aminohydroxycoumarin derivatives generally exhibited greater activity than the corresponding cyclic coumarin oxazin-2-one analogues 8 (Fig. 5), while an intramolecular hydrogen bond between the amino and hydroxyl groups was proposed to contribute to activity. Preliminary mechanistic studies implicated hemozoin inhibition and DNA interaction as possible contributors to the antiplasmodial effect.^68^

**Fig. 5:**
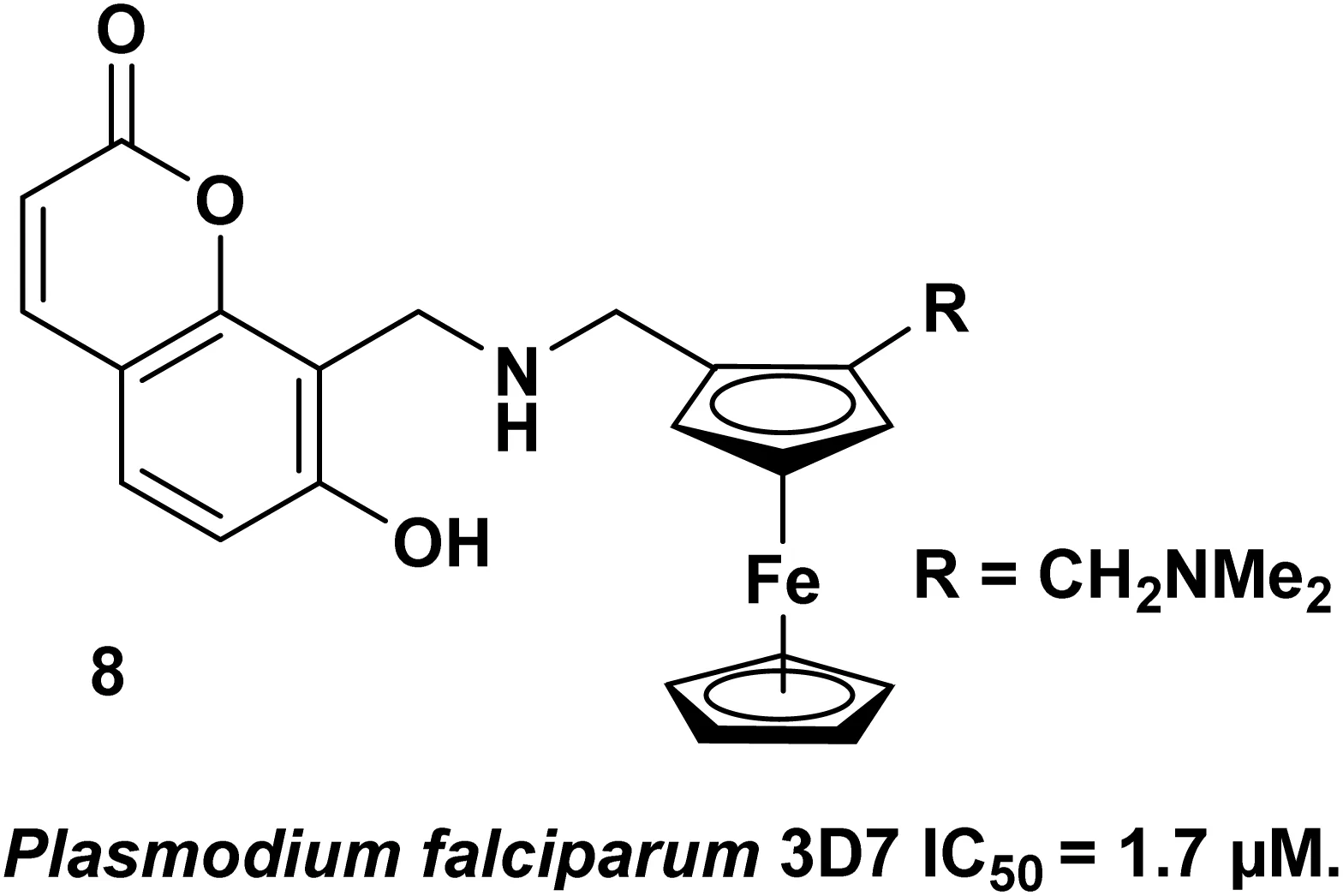
Chemical structure of a coumarin-based ferrocenyl 1,3-oxazine hybrid with antimalarial properties.

### 7.4 Antibacterial activities

Esfahani with the team synthesized coumarin–isoxazole–sulfonamide hybrids and evaluated their antibacterial activity against *Escherichia coli* and *Staphylococcus aureus*. Among the investigated derivative 9, bearing a para-methyl substituent on the sulfonamide-linked phenyl ring, showed the strongest experimental activity against *E. coli* (MIC = 32 µg mL^−1^) and an MIC of 128 µg mL^−1^ against *S. aureus* (Fig. 6). The 3D-QSAR model likewise identified 9f among the most active derivatives, with predicted pMIC values of 4.3 for both organisms, supporting its prioritization for antibacterial evaluation.^69^

**Fig. 6:**
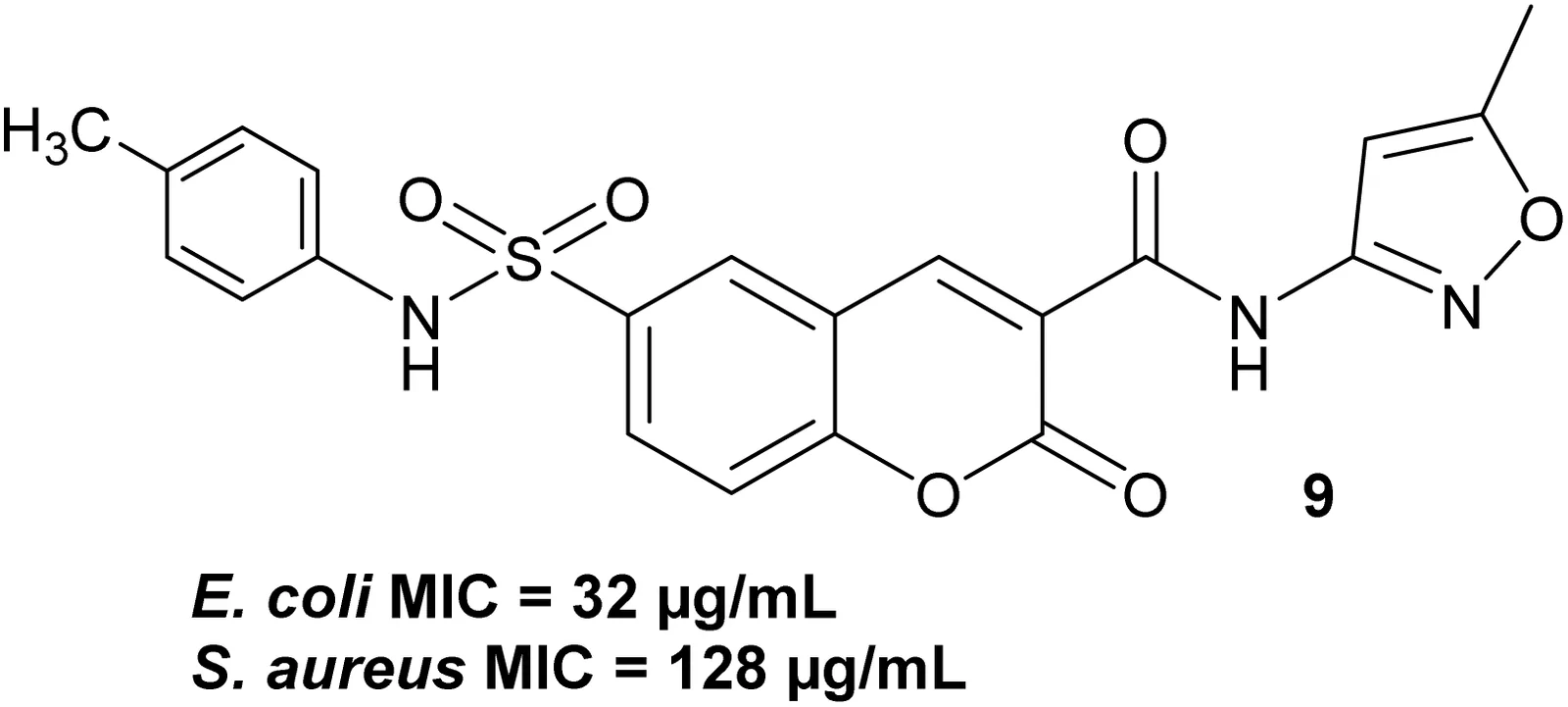
Structure of coumarin sulfonamide isoxazole hybrid with antibacterial activity.

Coumarins have also been investigated as anti-urease agents, particularly against *Helicobacter pylori*. Asghar *et al.* reviewed natural and synthetic coumarins and highlighted the influence of ring substitution on urease inhibition. One study reported that replacing the coumarin ester with a carboxylic acid, 2-(7-methoxy-2-oxo-2*H*-chromen-4-yl)acetic acid 10 (Fig. 7) produced strong antibacterial activity, with SAR identifying the carboxylic acid as critical for *anti-H. pylori* activity. Related coumarins lacking carboxylic acid or hydroxyl groups were inactive, whereas compounds bearing either functionality showed activity. Urease inhibition may therefore contribute to the antibacterial effect by interfering with the urease-dependent mechanisms that support *H. pylori* survival and colonization.^70^

**Fig. 7:**
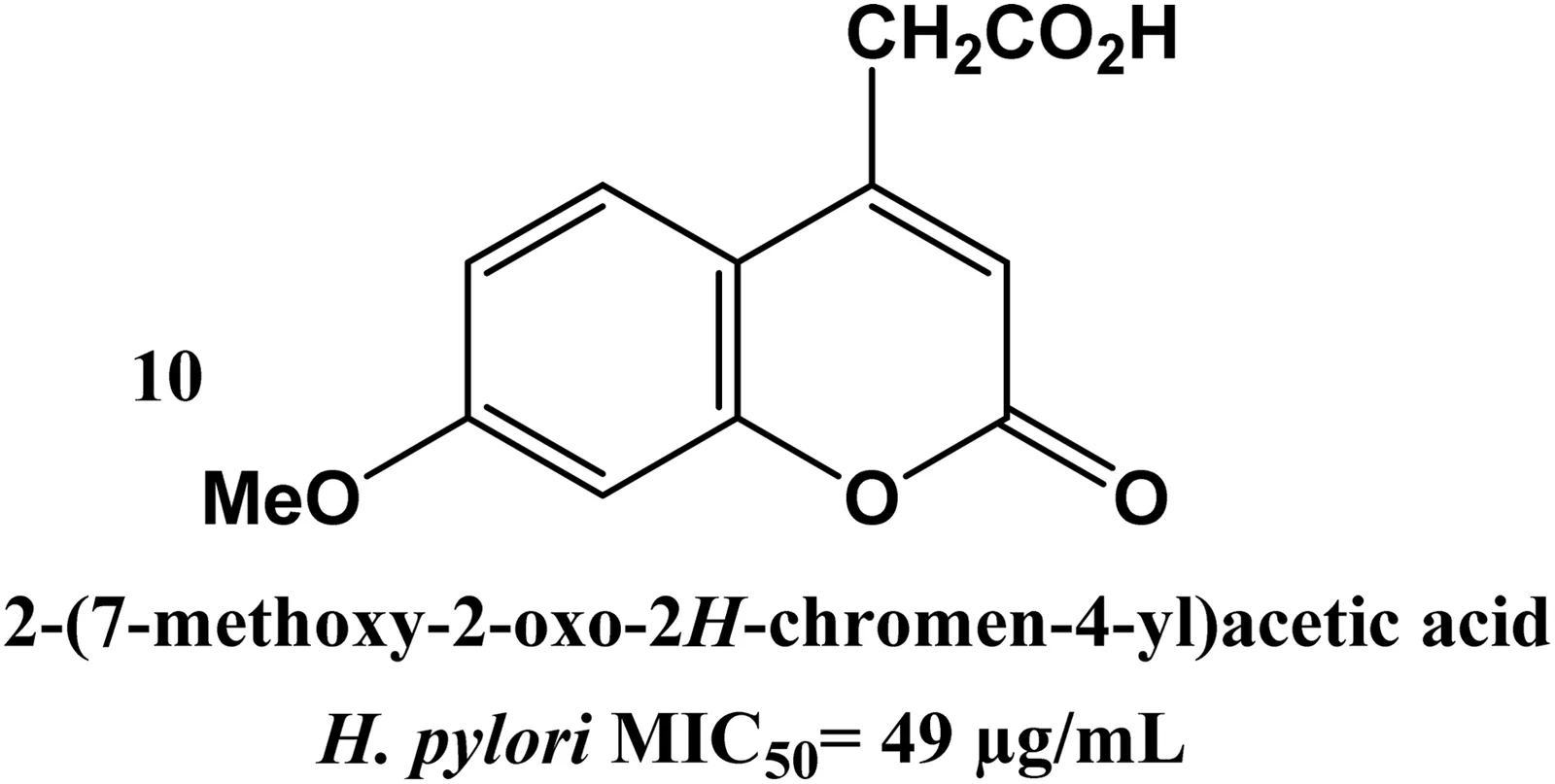
Coumarin derivatives with urease enzyme inhibitory activity.

A distinctive enzyme-inhibition mechanism has been established for carbonic anhydrase (CA). Supuran described coumarins as prodrug inhibitors that undergo CA-mediated hydrolysis of their lactone ring to generate 2-hydroxycinnamic acids, which bind at the entrance of the CA active site. This region exhibits substantial amino-acid variation among CA isoforms and is distinct from binding sites previously exploited by other inhibitor classes, providing a basis for the pronounced isoform selectivity of coumarin CA inhibitors. Structural and kinetic studies have therefore established coumarins 11 and 12 (Fig. 8) as a mechanistically distinct class of CA inhibitors. Because carbonic anhydrases are involved in maintaining pH and CO_2_/bicarbonate homeostasis, inhibition of bacterial CA may disrupt physiological processes required for microbial survival, providing a potential basis for antibacterial activity.^71^

**Fig. 8:**
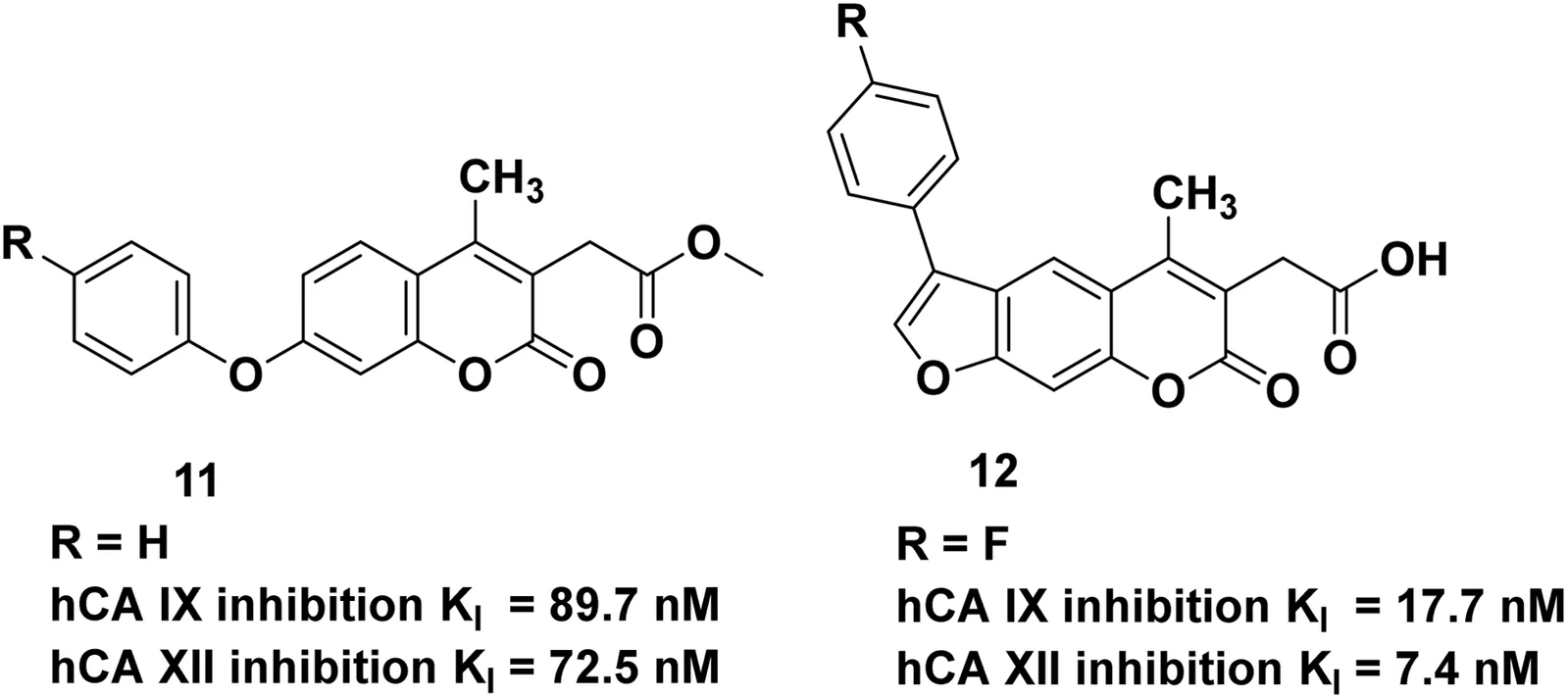
Coumarin derivatives with carbonic anhydrase enzyme inhibitory activity.

Building on this, Giovannuzzi and co-researchers extended coumarin CA inhibition to bacterial targets by evaluating fourteen simple coumarins against the α-class CAs NgCAα from *Neisseria gonorrhoeae* and VchCAα from *Vibrio cholerae*. NgCAα was inhibited with *K*_i_ values of 28.6–469.5 µM, while VchCAα showed *K*_i_ values of 39.8–438.7 µM. In comparison, the human isoforms hCA I and hCA II were less susceptible, with *K*_i_ ranges of 137–948.9 µM and 296.5–961.2 µM, respectively. Compound 13 is the most potent NgCAα inhibitor, 4-methyl-7-diethylaminocoumarin (Fig. 9), had a *K*_i_ of 28.6 µM. Substituent nature and position strongly influenced activity, with the study providing evidence that coumarins can inhibit bacterial CAs while offering selectivity over human CA I and II. This preferential inhibition of bacterial carbonic anhydrases provides a potential mechanistic basis for the antibacterial activity of coumarin derivatives.^72^

**Fig. 9:**
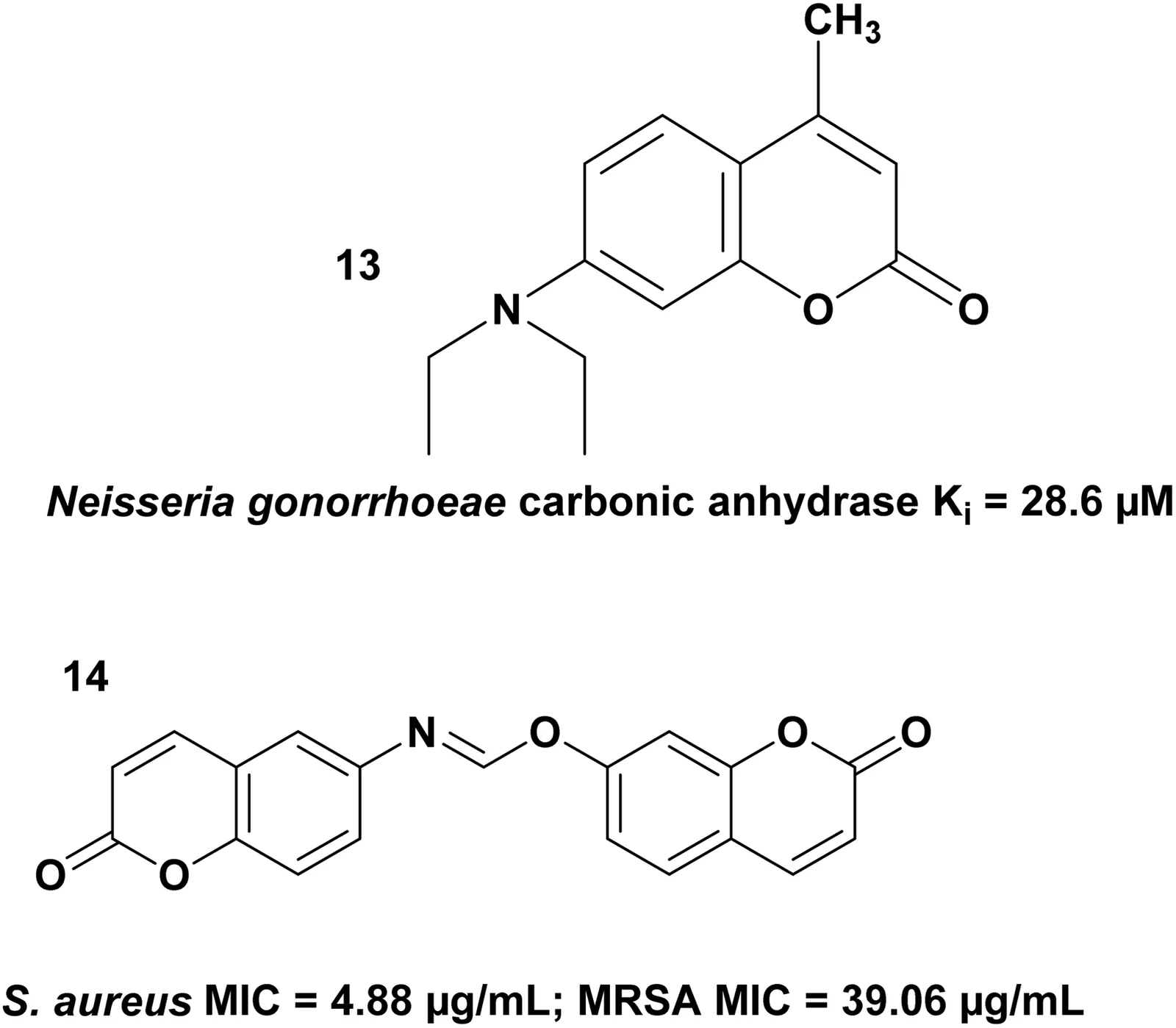
Coumarin derivative with *Neisseria gonorrhoeae* carbonic anhydrase enzyme inhibitory activity and antibacterial activity.

More recently, Ahmed *et al.* synthesized a series of coumarin-based heterocyclic derivatives and evaluated their antimicrobial and antibiofilm activities. Among the investigated compounds, compound 14 (Fig. 9) showed the strongest antibacterial activity against *S. aureus,* with MIC and MBC values of 4.88 and 9.76 µg mL^−1^, respectively, compared with the 19.53 and 39.06 µg mL^−1^ for neomycin under the same assay conditions. The compound also inhibited MRSA, although at higher concentrations (MIC = 39.06 µg mL; MBC = 76.7 µg mL^−1^), while activity against *E. coli* was weaker (MIC = 78.13 µg mL^−1^). These indicate greater activity against the investigated Gram-positive strains and further demonstrate that structural modification of the coumarin scaffold can influence planktonic growth.^73^

### 7. 5Antifungal activities

Yang *et al.* synthesized a series of coumarin–thiazole derivatives and evaluated their activity against *Fusarium moniliforme*, *F. graminearum*, and *Curvularia lunata*. Compound 15 showed the highest inhibition against *F. moniliforme* (74% at 0.5 mg mL^−1^), whereas 16 was the most active against *F. graminearum* (89%) and *C. lunata* (93.4%) at the same concentration (Fig. 10). Incorporation of a trifluoromethyl group was associated with enhanced antifungal activity, with 16 showing greater activity than its corresponding non-trifluoromethyl analogue.^74^

**Fig. 10:**
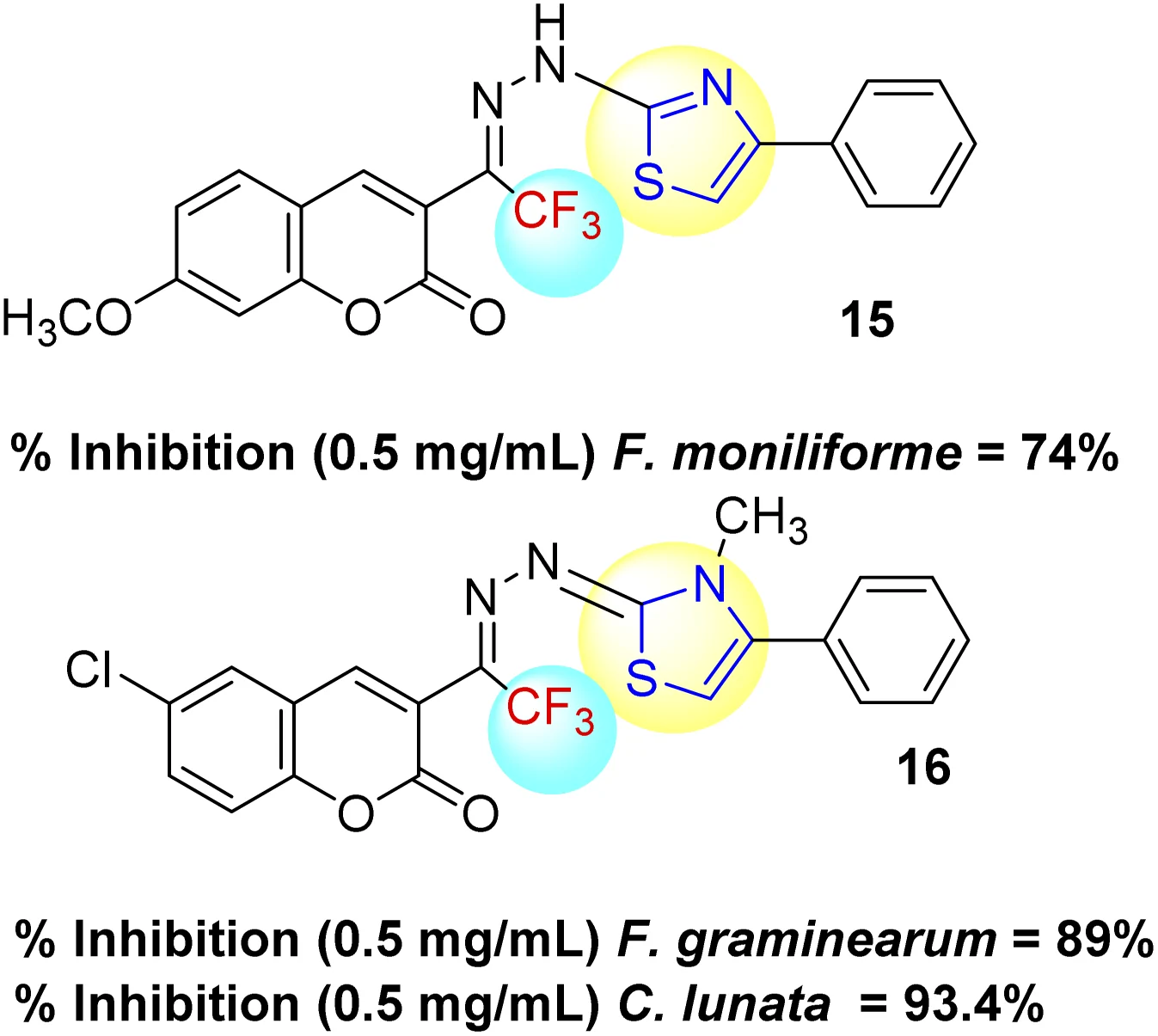
(Coumarin-3-yl) trifluoroethylidene thiazole derivatives with antifungal activities.

### 7. 6Antitubercular activities

Rahul and Rahul synthesized a 9-compound series of 5-(substituted) aryl-3-(3-coumarinyl)-1-phenyl-2-pyrazolines and evaluated their activity against *Mycobacterium tuberculosis* H37Rv using the Alamar Blue assay. The incorporation of an aminobenzoic acid moiety was associated with improved antitubercular activity across the salicylaldehyde-, benzaldehyde-, and cinnamaldehyde-derived series. Among the compounds 17, the salicylaldehyde-derived analogue bearing the aminobenzoic acid substituent, showed the highest activity, with a MIC of 0.8 µg mL^−1^ (Fig. 11), compared with 3.125 µg mL^−1^ for streptomycin under the reported assay conditions.^75^

**Fig. 11:**
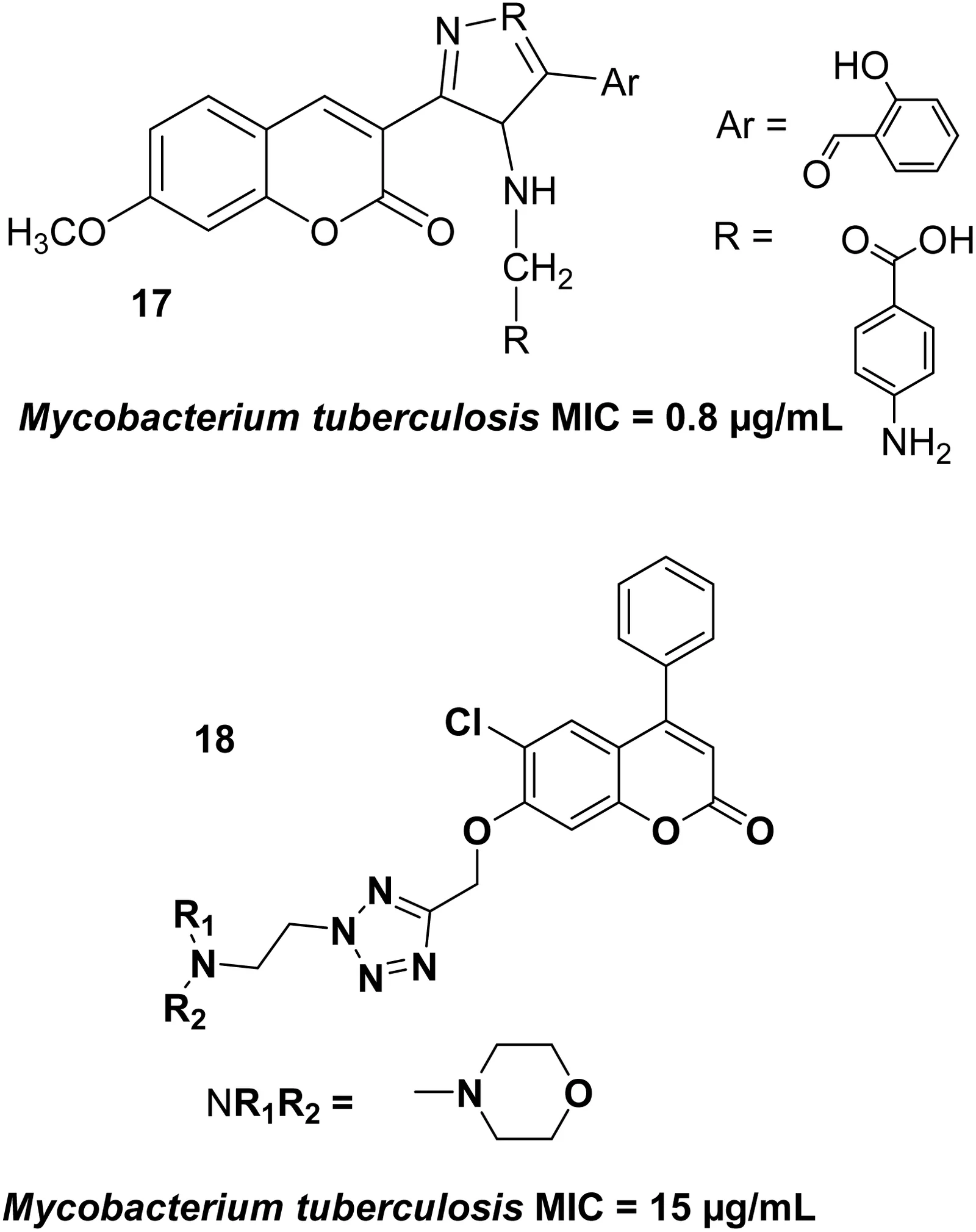
5-(Substituted) aryl-3-(3-coumarinyl)-1-phenyl-2-pyrazolines and tetrazole derivative coumarin with antitubercular activities.

More recently, Batran *et al.* synthesized a series of azaheterocyclic coumarin derivatives and evaluated their antitubercular activity against *M. tuberculosis* H37Rv. Among the synthesized derivatives, the tetrazole-containing compound 18 had the highest activity with an MIC of 15 µg mL^−1^, and retained activity against the ΔkatG mutant strain. Compound 18 also penetrated *M. tuberculosis*-infected human macrophages and suppressed intracellular bacillary growth. Enzyme assays identified 18 as an inhibitor of enoyl-acyl carrier protein reductase (InhA), with an IC_50_ of 0.565 µM. SAR analysis also indicated that incorporating the tetrazole moiety into the 4-phenylcoumarin framework highly favored antitubercular activity.^76^

### 7.7 Antileishmanial activities

Cuéllar *et al.* evaluated the *in vitro* antileishmanial activity of 13 coumarin–chalcone hybrids against the intracellular amastigote stage of *Leishmania panamensis* using the MTT microassay, with amphotericin B as the reference drug. Among the synthesized compounds 19a, bearing a 3-hydroxyphenyl substituent, showed the highest activity against *L. panamensis* amastigotes (EC_50_ = 2.1 ± 0.1 µM) (Fig. 12), followed by 19b, bearing a 3-methoxyphenyl substituent (2.5 ± 0.2 µM), and 19c, bearing an unsubstituted phenyl group (3.7 ± 0.5 µM).^77^

**Fig. 12:**
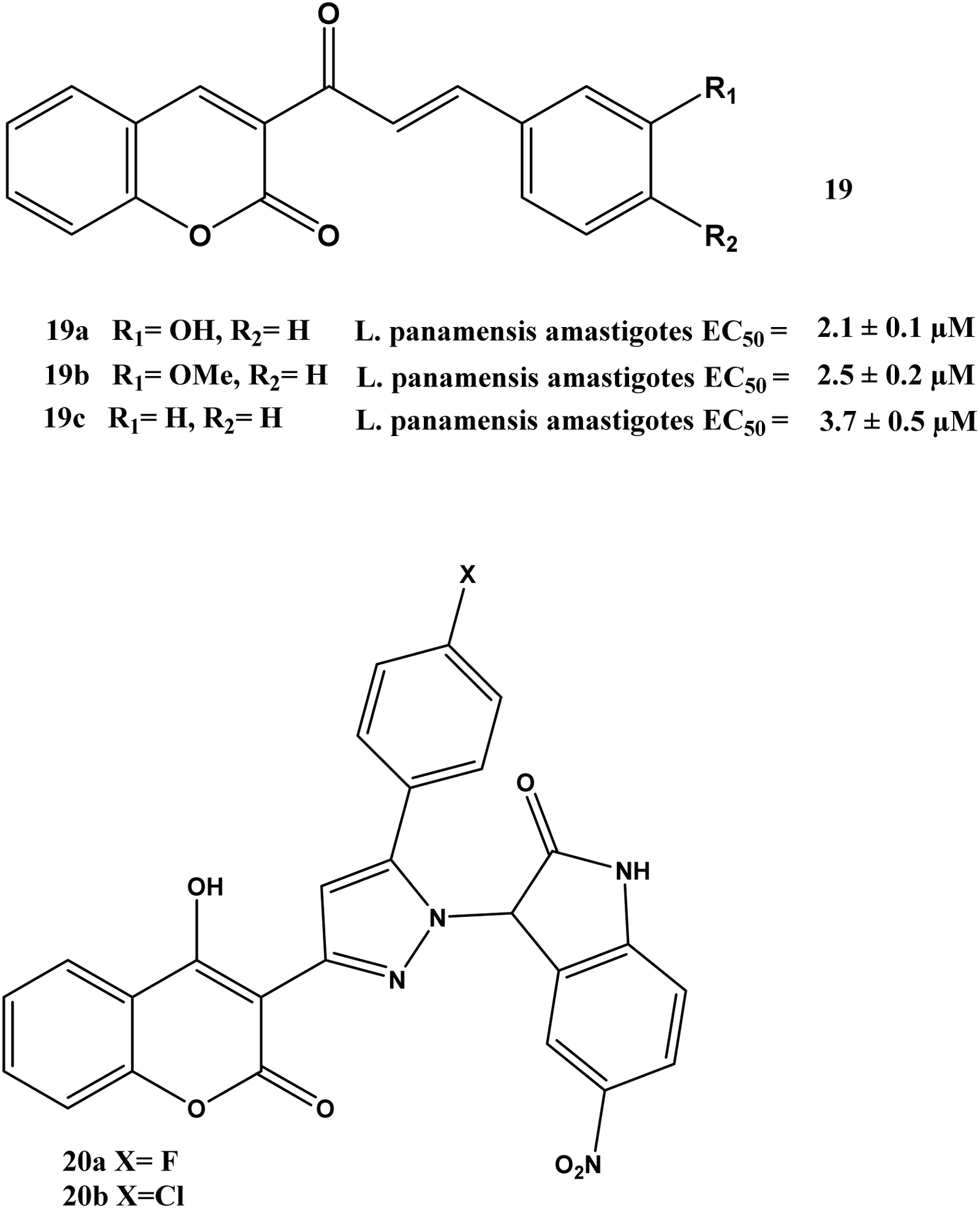
Representative coumarin-chalcone and coumarinyl-indolinone derivative with anti-Leishmanial activity.

A series of fifteen coumarinyl-indolinone hybrids incorporating 4-hydroxycoumarin and pyrazolyl indolin-2-one motifs as synthesized and evaluated against *Leishmania major* by Batran *et al.* Compounds 20a and 20b (Fig. 12) were the most active derivatives, with IC_50_ values of 1.21 ± 0.18 and 1.43 ± 0.26 µM, respectively, against promastigotes and 2.41 ± 0.22 and 3.78 ± 0.28 µM against intracellular amastigotes. Under the same conditions, the reference drug miltefosine showed IC_50_ values of 7.83 ± 0.34 and 8.07 ± 0.24 µM against the respective parasite forms.^78^

### 7.8 Antiviral activities

Coumarin derivatives have also been explored against viral pathogens, including HIV-1 and SARS-CoV-2.^93^ Foboufu and co-authors isolated the previously undescribed dimeric coumarin bichromonol from the stem bark of *Hypericum roeperianum* and evaluated its *anti*-HIV activity in infected MT-4 cells. Bichromonol 21 showed an EC_50_ of 8.7 µM against HIV-1_IIIB_ and 6.6–12.0 µM against the tested resistant variants, with its strongest relative activity observed against the A17 and EFVᴿ variants, for which EC_50_ values of 8.3 and 6.6 µM, respectively, were more active than that of nevirapine (80 and 100 µM) (Fig. 13). Its CC_50_ of 54 µM in MT-4 cells was also relatively improved than that of efavirenz (40 µM).^79^

**Fig. 13:**
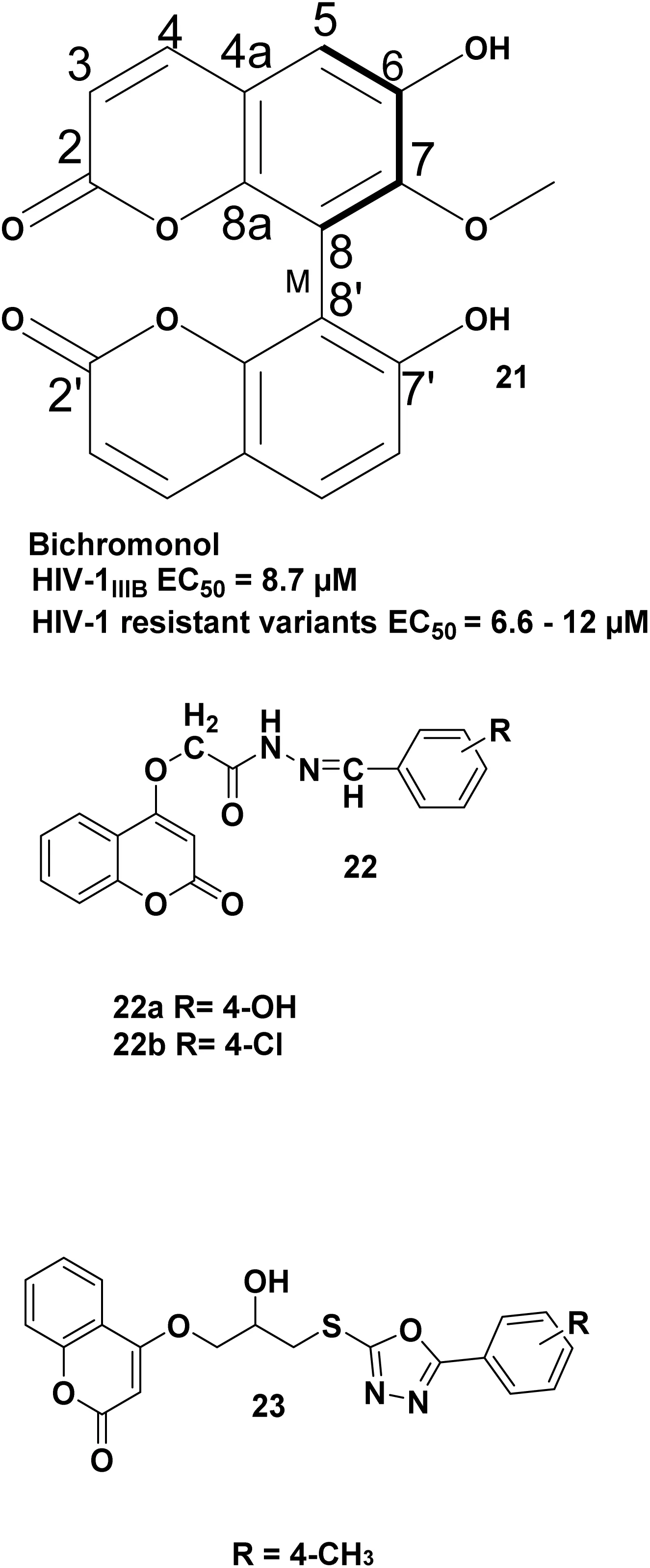
Structure of coumarin derivatives with antiviral properties.

Sa'ad *et al.* synthesized a series of coumarin analogues incorporating Schiff-base and thiazolidinone functionalities and investigated their antiviral activity against coronaviruses. Compounds 22a and 22b (Fig. 13) showed the strongest activity against human coronavirus OC43(HCoV-OC43), with IC_50_ values of 39.8 and 41 µg mL^−1^, respectively. Compound 22a also exhibited a comparatively high CC50 value of 500.55 µg mL^−1^ in HEK-293 cells.^80^ In another study, coumarin derivatives containing 1,3,4-oxadiazole or 1,3,4-thiadiazole moieties were synthesized and evaluated against tobacco mosaic virus. Among the derivatives, Compound 23 displayed therapeutic and protective EC_50_ values of 180.7 and 190.3 µg mL^−1^, respectively, when compared with 284.1 and 247.1 µg mL^−1^ for reference Ningnanmycin^81^

### 7. 9Anticancer and antiproliferative activities

Rawat and Reddy reviewed the anticancer potential of coumarin derivatives, highlighting synthetic coumarins containing azoles, sulfonyl, furan, and pyrazole motifs and their reported anticancer, antitumour, and antiproliferative activities. These effects have been associated with diverse mechanisms, including apoptosis induction, cell-cycle arrest, tubulin/microtubule disruption, and oxidative stress modulation.^94–96^ Beyond direct cytotoxicity, the redox and photophysical properties of coumarins have also enabled their development as chemotherapy adjuvants and mechanistic probes.^82,84^

Patra *et al.* demonstrated the potential of coumarin as a chemotherapy adjuvant by developing methylumbelliferone selenocyanate (MUS) 24, an organoselenium coumarin derivative (Fig. 14), as a myeloprotective adjunct to carboplatin. In female Swiss albino mice, oral MUS (6 mg kg^−1^) administered alongside or before carboplatin (12 mg kg^−1^, i.p.) reduced chromosomal aberrations, micronucleus formation, DNA damage, and apoptosis in bone marrow cells while restoring bone marrow cellularity. This protection was associated with suppression of reactive oxygen and nitrogen species and restoration of the glutathione redox balance. Importantly, MUS did not compromise anticancer activity; instead, it enhanced carboplatin cytotoxicity against MCF-7 and Colo-205 cells *in vitro* and potentiated its antitumor effects in Dalton's lymphoma. This approach exploits the redox activity of the coumarin derivative to protect normal tissue while simultaneously enhancing the activity of an established cytotoxic agent.^82^

**Fig. 14:**
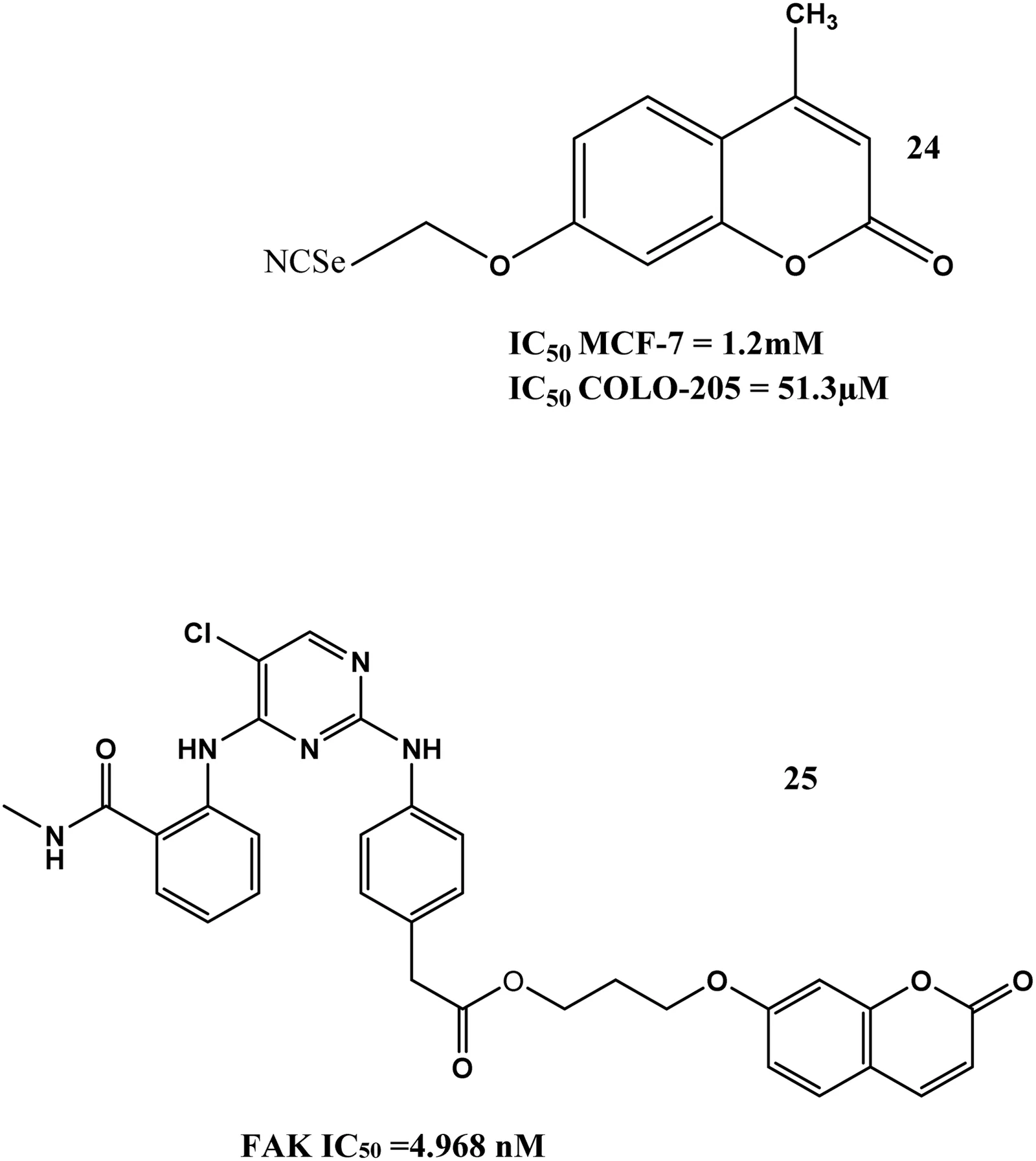
Structure of an organoselenium coumarin and the 2,4-diphenylpyrimidine derivative with anti-tumor activities.

A series of coumarin-containing 2,2-diphenylpyrimidine derivatives was synthesized and evaluated as focal adhesion kinase (FAK) inhibitors against non-small-cell lung cancer (NSCLC). Compound 25 emerged as the most active derivative, exhibiting IC_50_ of 0.28 µM against H1299 cells and potent inhibition of FAK (IC_50_ = 4.968 nM). The compound also inhibited cancer-cell migration and invasion, induced cell-cycle arrest and apoptosis and reduced MMP-2 and MMP-9 expression. *In vivo* evaluation also demonstrated inhibition of implanted NSCLS tumor growth in mice, and it was reported to be relatively well tolerated^83^

A further extension of the use coumarin is its incorporation into anticancer compounds as a mechanistic probe. Schroeder *et al.* introduced a coumarin moiety into schweinfurthin 26 (Fig. 15), a naturally occurring stilbene with potent, selective antiproliferative activity but an incompletely defined mechanism, with oxysterol-binding proteins and *trans*-Golgi trafficking proposed as potential targets. The resulting analogue retained significant antiproliferative activity across the NCI-60 panel, particularly against glioblastoma, leukaemia, and renal cancer cell lines, though ovarian lines remained resistant. Coumarin incorporation additionally conferred fluorescence (excitation ∼420 nm; emission ∼590 nm), enabling the analogue to serve as a probe for cellular localisation and future mechanism of action studies.^97^

**Fig. 15:**
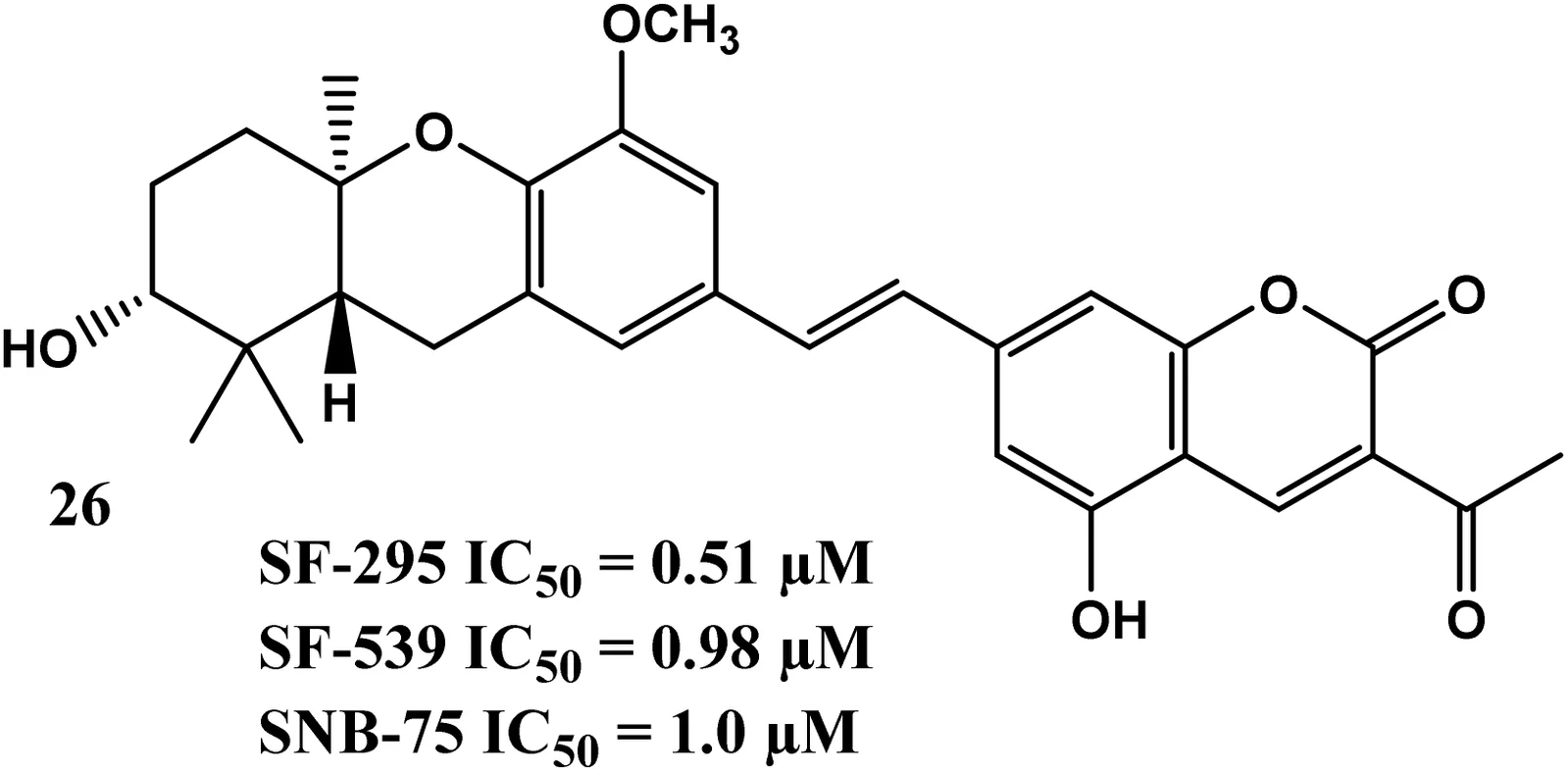
Fluorescent coumarin Schweinfurthin analog with anti-proliferative activities.

### 7.10 Anti-inflammatory and analgesic activities

Adsule *et al.* synthesised coumarin-acetohydrazide–isatin hybrids 27 by condensing substituted isatins with 7-hydroxy-4-methylcoumarin and assessed their anti-inflammatory activity. The derivatives were evaluated using molecular docking against COX and the carrageenan-induced paw oedema model in rats at 200 mg kg^−1^. The 5-nitro-isatin and 1-methyl-isatin derivatives (Fig. 16) produced the highest activity, achieving 71.94–77.96% inhibition after 5 h, exceeding the activity of celecoxib at the same time point. Structure–activity analysis indicated that substitution on the isatin ring strongly influenced anti-inflammatory activity, with electron-withdrawing substituents such as nitro and chloro favouring activity. Molecular docking further supported interactions with COX, providing a structural basis for the observed activity.^85,98^

**Fig. 16:**
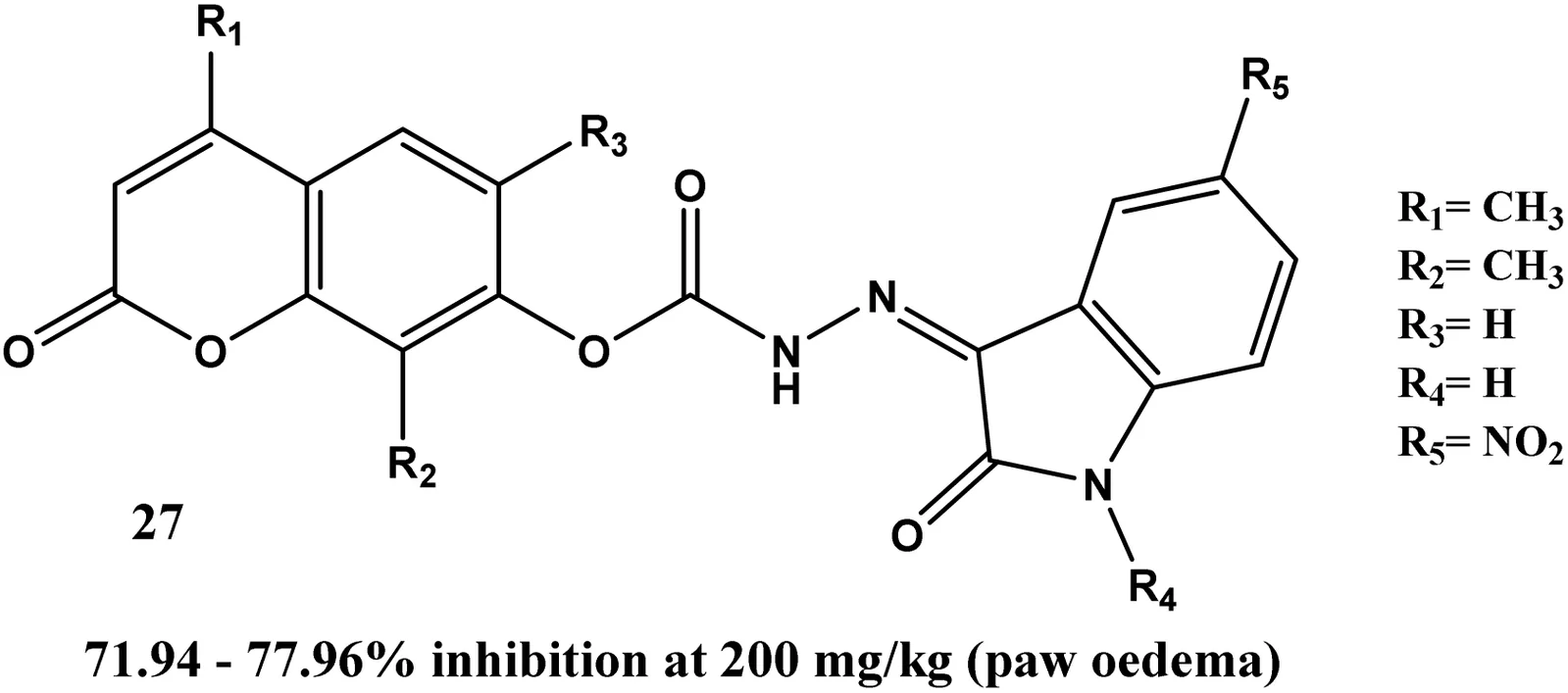
Coumarin-acetohydrazide–isatin hybrids with anti-inflammatory activities.

Srivastava *et al.* investigated a different strategy by synthesising 7-substituted coumarin–benzothiazole derivatives 28 (Fig. 17) and evaluating their analgesic activities alongside ulcerogenic risk. The most active compounds were subsequently assessed for 5-lipoxygenase (5-LOX) inhibition. Compounds 28c and 28e showed the strongest overall activity, with compound 22e exhibiting potent 5-LOX inhibition (IC_50_ = 2.118 ± 0.024 nM) and a mixed/non-competitive inhibition pattern. Both compounds also produced fewer gastric lesions than acetylsalicylic acid, indicating a more favourable ulcerogenic profile. The 5-LOX data showed a preference for –Cl or –OCH_3_ substitution at the C6 position of the benzothiazole ring, while additional substitution reduced inhibitory potency.^86^

**Fig. 17:**
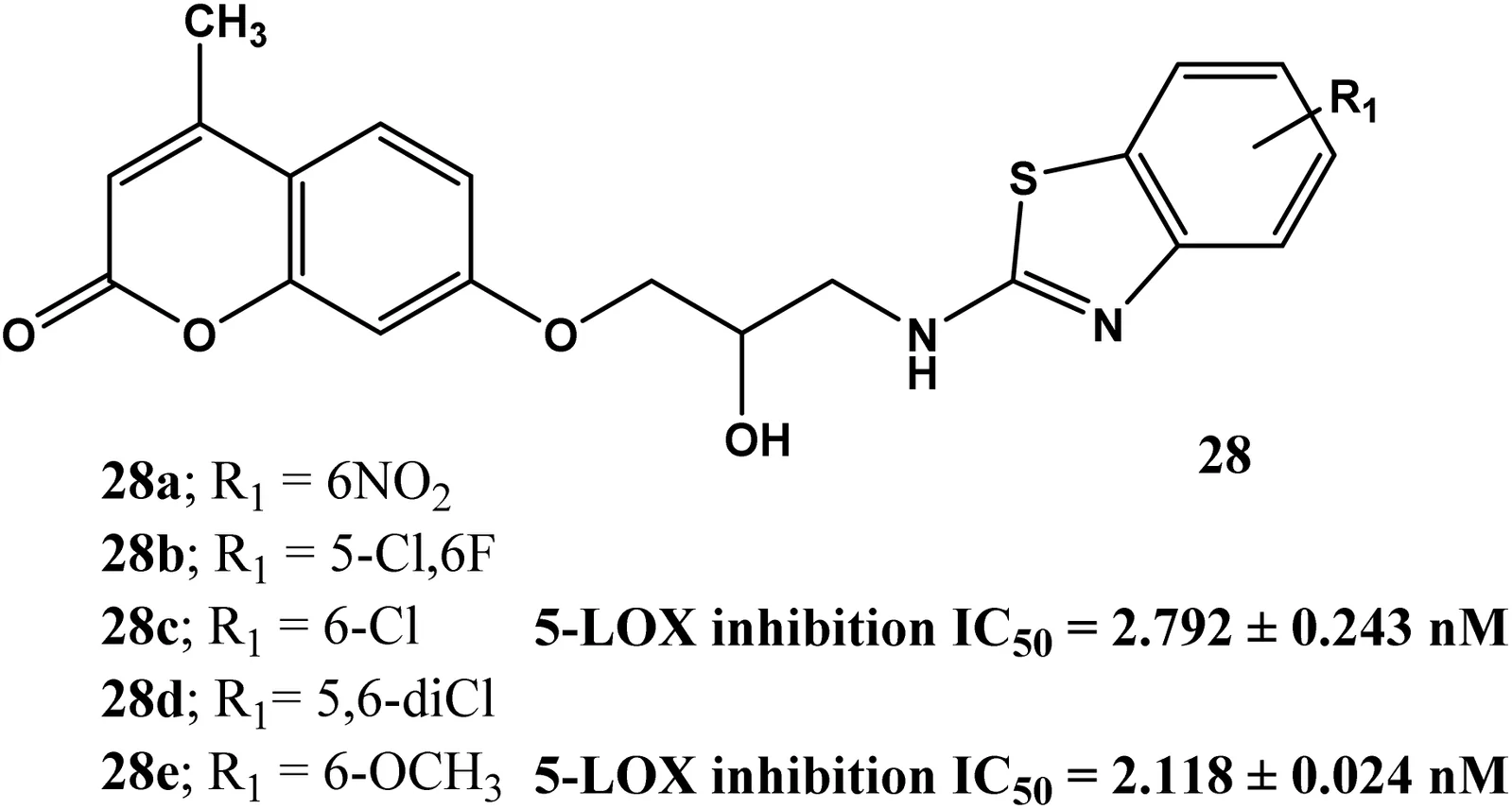
7-Substituted coumarin–benzothiazole derivatives with analgesic activities.

### 7.11 Neurological and neuroprotective activities

Zhou *et al.* explored coumarin as a scaffold for serotonergic antidepressant development by synthesising 7-(2-(4-substituted-piperazin-1-yl)-2-oxoethoxy)-2*H*-chromen-2-ones 29, incorporating a piperazine moiety to facilitate interaction with serotonergic targets. Antidepressant activity was evaluated using the forced swim and tail suspension tests, while the lead compound was further assessed in the open-field test to exclude confounding psychostimulant effects and by brain 5-HT quantification. The most active derivative, 7-(2-(4-(4-fluorobenzyl)piperazin-1-yl)-2-oxoethoxy)-2*H*-chromen-2-one (29), significantly reduced immobility measured by difference in mobility duration (DID) at 40 mg kg^−1^ (0.01 < *P* < 0.05; DID = 26.09) (Fig. 18), comparable to fluoxetine (0.01 < *P* < 0.05; DID = 28.80). The compound also increased brain 5-HT levels and showed favourable blood–brain barrier permeability. Molecular docking indicated strong interactions within the 5-HT1A receptor active site, supporting receptor engagement as a potential contributor to the observed activity. SAR analysis identified the 4-fluorobenzyl-piperazine substituent at the coumarin 7-position as optimal, highlighting the contribution of both the piperazine linker and terminal aryl group to activity.^48^

**Fig. 18:**
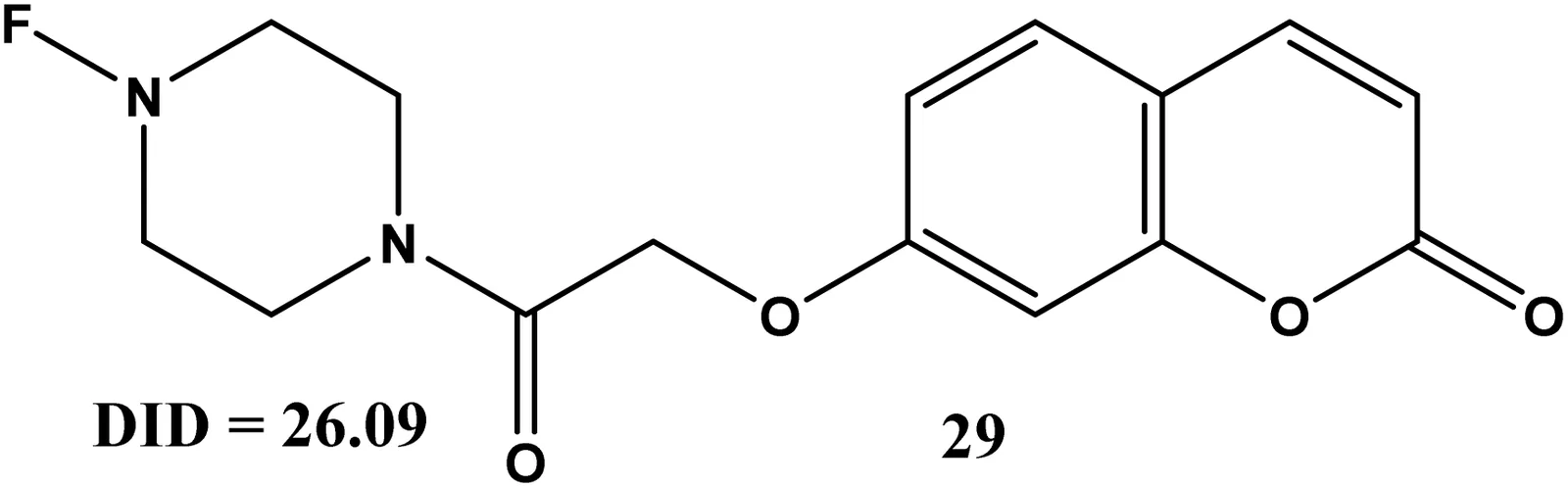
7-(2-(4-(4-Fluorobenzyl)piperazin-1-yl)-2-oxoethoxy)-2*H*-chromen-2-one with neurological and neuroprotective activities.

Coumarin has also been incorporated into multi-target-directed ligand (MTDL) designs for Alzheimer's disease (AD), where simultaneous modulation of cholinergic dysfunction, oxidative stress, and neuroinflammation is desirable.^87,99^ Two series of coumarin–1,3,4-oxadiazole hybrids were evaluated for acetylcholinesterase (AChE), butyrylcholinesterase (BuChE), antioxidant, and cyclooxygenase (COX) inhibitory activities (Fig. 19). Two compounds showed the strongest AChE inhibition (IC_50_ = 29.56 and 28.68 µM, respectively), with selectivity indices of 1.552 and 1.652, exceeding that of galantamine (1.132), while compound 30 was the most potent BuChE inhibitor (IC_50_ = 23.97 µM). Their concurrent antioxidant and anti-inflammatory activities further supported the intended multi-target pharmacological profile.^24,100^

**Fig. 19:**
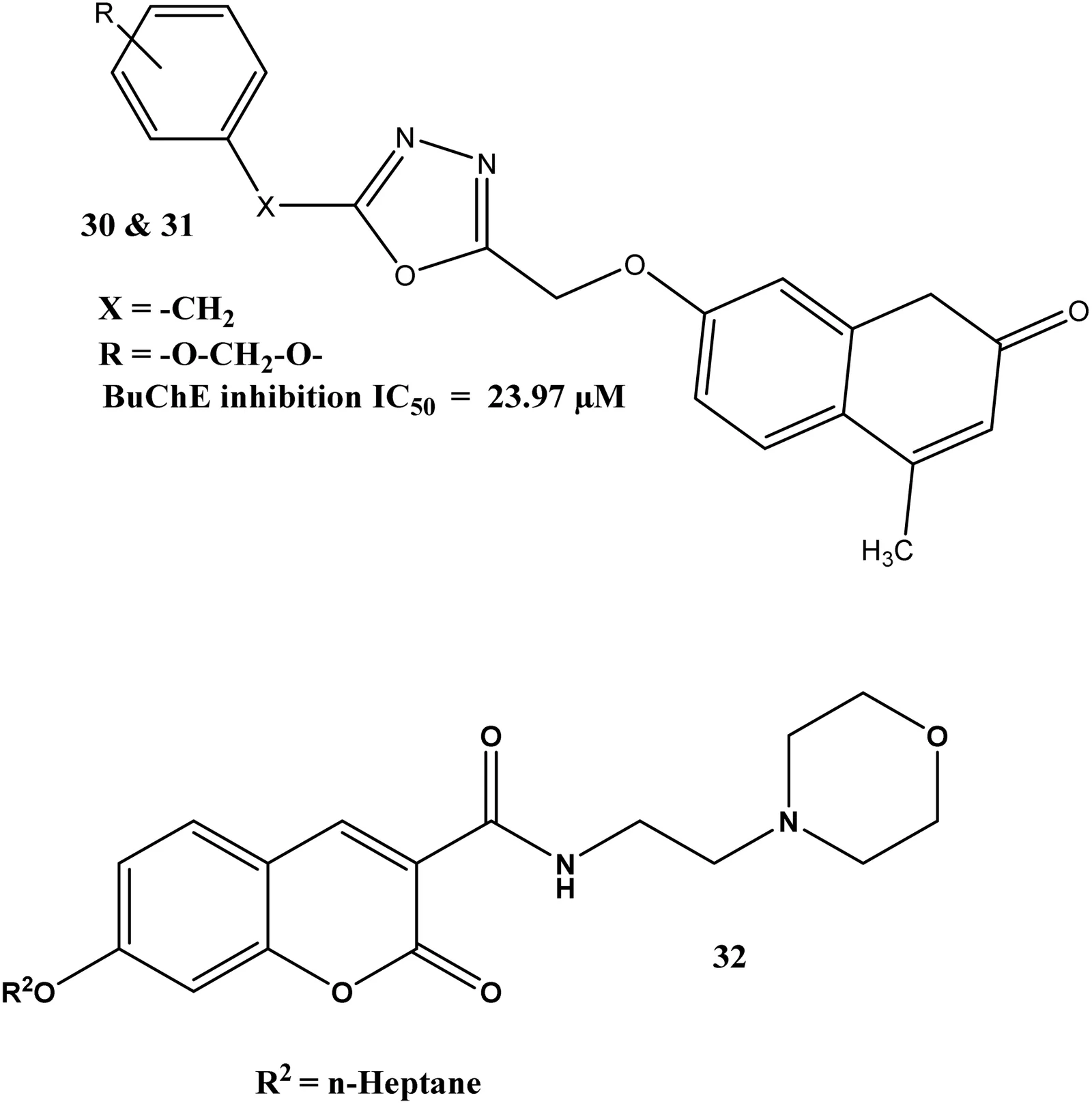
Structure of 1,3,4-oxadiazole and phenolic acid hybrids as anti-Alzheimer's agents and morpholine-coumarin derivatives for inflammation-associated depression.

Liu *et al.* recently developed morpholine-coumarin derivatives as dual inhibitors of indoleamine 2,3-dioxygenase 1(IDO1) and tryptophan 2,3-dioxygenase (TDO) for inflammation-associated depression. Compound 32 showed the highest dual inhibitory activity, with IC50 values of 0.34 ± 0.06 and 0.75 ± 0.10 µM against IDO1 and TDO, respectively. In LPS-stimulated BV2 microglial cells, 32 downregulated IDO1/TDO expression and reduced pro-inflammatory mediators including IL-1β, COX-2, iNOS and TNF-α, while increasing IL-10. Pharmacokinetic evaluation in mice following an intraperitoneal administration at 20 mg kg^−1^ showed a half-life of 22.33 ± 5.11 h and bioavailability at 73%. Also, at the same dose 32 reduced immobility in the forced-swim and tail-suspension test in an LPS-induced acute depressive mouse model without affecting spontaneous locomotor activity^88^

### 7.12 Antidiabetic activity

Coumarin-based anti-diabetic activity has been described through two principal mechanisms: inhibition of intestinal carbohydrate-digesting enzymes, which limits post-prandial glucose absorption, and suppression of hepatic gluconeogenesis, which reduces endogenous glucose production.^89^ Ranđelović and Bipat reviewed coumarin and related compounds (Fig. 20) across these mechanisms scopoletin 33, decursinol 34, and coumarin–flavonoid hybrids were reported as α-glucosidase inhibitors, delaying carbohydrate hydrolysis and glucose uptake, similar to acarbose. Other derivatives, including coumarin, umbelliferone, and coumarin-3-carboxylic acids, were reported to suppress gluconeogenesis, indicating effects on hepatic glucose production.^89^

**Fig. 20:**
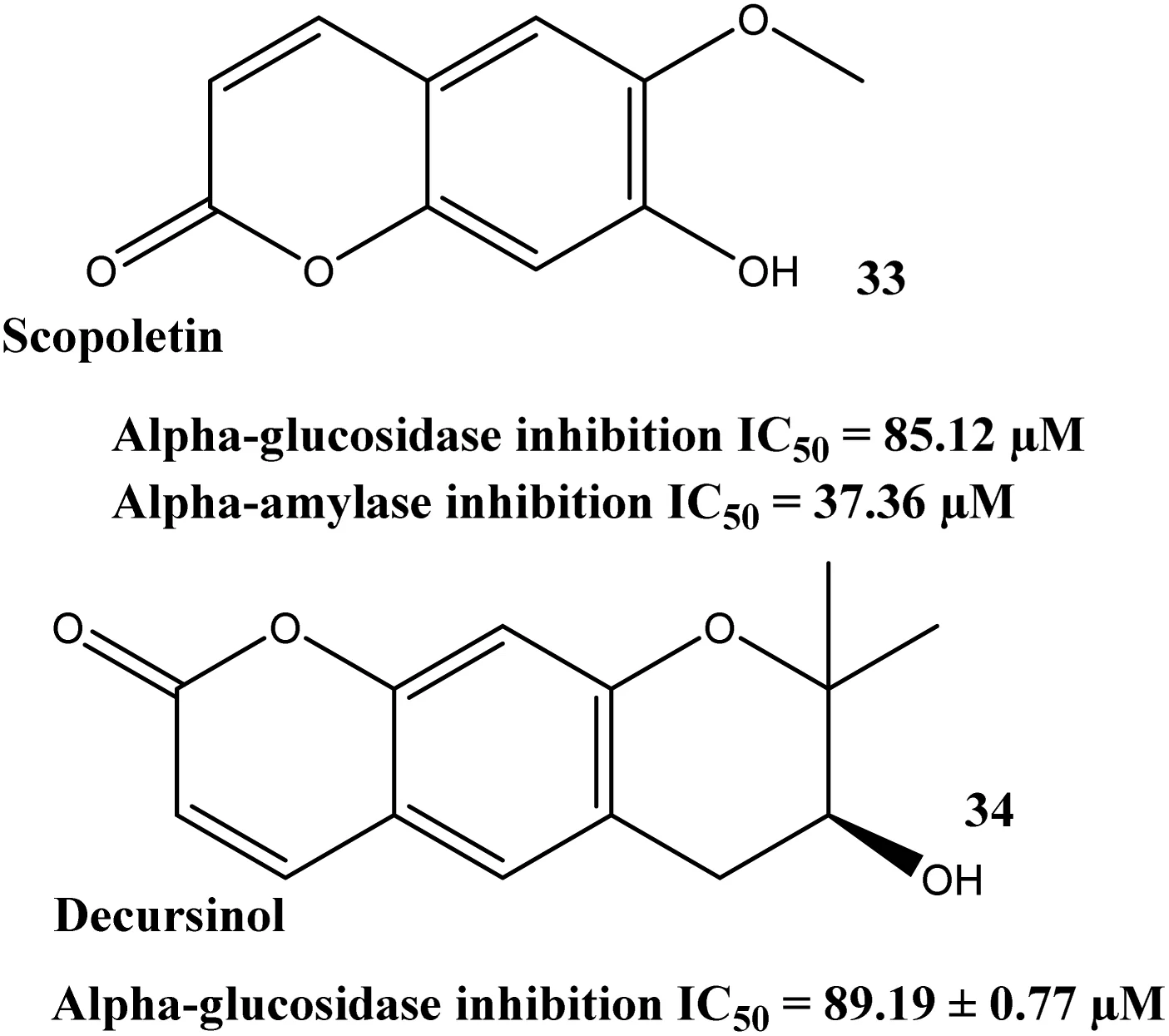
Antidiabetic coumarin compounds.

Providing additional evidence for the enzyme-inhibition approach, Patagar *et al.* in 2023 synthesized benzimidazole-tethered coumarin-3-carboxamides and evaluated their antioxidant activity using DPPH and ABTS assays and their α-amylase inhibitory activity using the DNSA method. Compound 35, bearing a 3,5-dimethoxyphenyl carboxamide substituent (Fig. 21), showed the strongest α-amylase inhibition in the series (IC_50_ = 67.52 µM), compared with metformin (IC_50_ = 54.13 µM). Molecular docking against α-amylase (PDB 4X9Y) further supported direct enzyme binding. SAR analysis identified the 3,5-dimethoxyphenyl substituent as optimal for both α-amylase inhibition.^90^

**Fig. 21:**
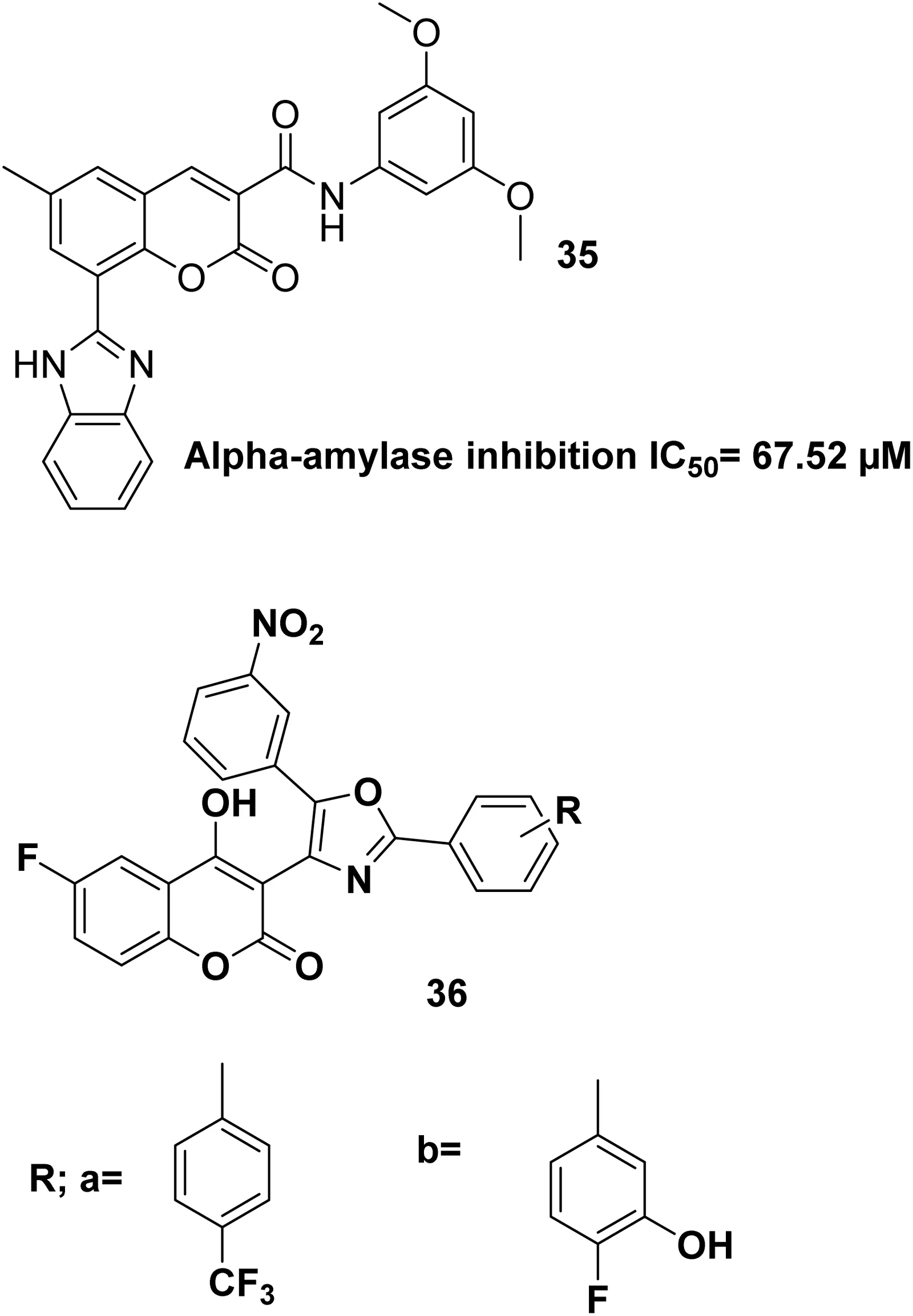
Benzimidazole-tethered coumarin-3-carboxamides and coumarin-oxazole derivatives with antidiabetic activity.

Hussien *et al.* synthesized a series of sixteen coumarin-oxazole hybrids and evaluated their inhibitory activity against α-amylase and α-glucosidase. Compound 36a (Fig. 21), bearing a *para*-trifluoromethyl substituent on the terminal aromatic ring was the most active derivative having IC_50_ values of 0.70 ± 0.05 µM against α-amylase and 1.10 ± 0.10 µM against α-glucosidase. Compound 36b (Fig. 21), containing 3-hydroxy and 4-fluoro substituents also showed potent inhibition with corresponding IC_50_ values of 0.90 ± 0.05 and 1.20 ± 0.10 µM. Under the same conditions, acarbose had IC_50_ values of 10.30 ± 0.20 and 9.80 ± 0.20 µM respectively.^101^

### 7.13 Antioxidant activities

Alshibl *et al.* synthesised pyranocoumarin, coumarin-3-sulfonamide, and coumarin-sulfonamide chalcone derivatives and evaluated their radical-scavenging activity using the DPPH assay, with ascorbic acid as the reference standard. 4-Hydroxy-6-methylcoumarin and 6-chloro-4-hydroxycoumarin were selected as pharmacophoric moieties. Pyranocoumarins were largely inactive (IC_50_ > 200 µg mL^−1^), whereas the coumarin-3-sulfonamides derived from sulfa-drugs (sulfanilamide/sulfadiazine/sulfathiazole) showed moderate activity (IC_50_ = 61.78–120.12 µg mL^−1^). However, the subsequent aniline-derived coumarin-3-sulfonamides series 37 (Fig. 22) with substituted phenyl groups showed a markedly improved activity (IC_50_ = 3.87–35.36 µg mL^−1^). Compounds 37c and 37d in this series, bearing a 4-hydroxyphenyl substituent, were the most potent (IC_50_ = 3.87 and 4.30 µg mL^−1^, respectively), comparable with ascorbic acid (2.83 µg mL^−1^). 6-Chloro substitution generally favoured activity over the corresponding 6-methyl substitution. In contrast, the coumarin-sulfonamide chalcones were inactive (IC_50_ > 200 µg mL^−1^), indicating that extension of the scaffold through a chalcone linker did not improve radical-scavenging activity.^91^

**Fig. 22:**
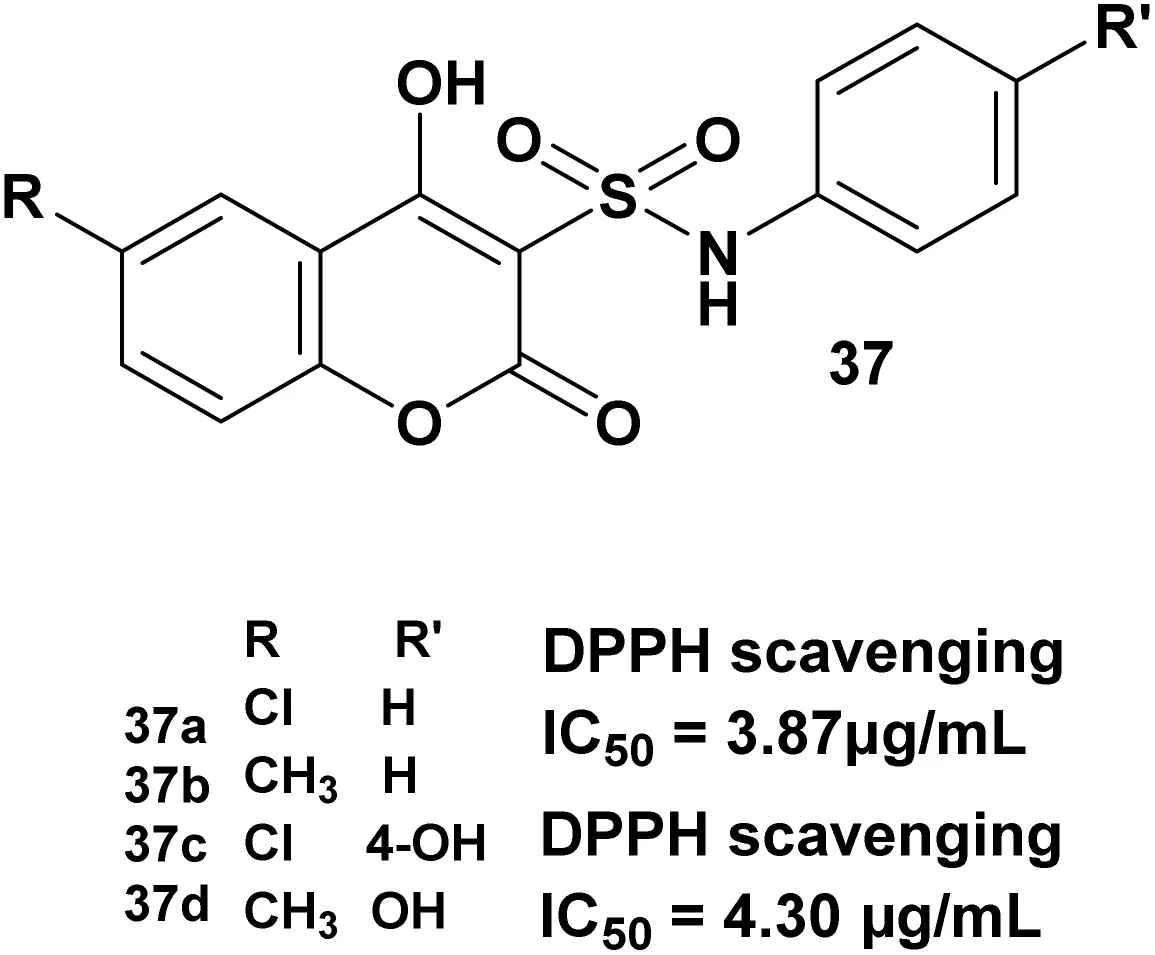
Coumarin-3-sulfonamide derivatives with antioxidant activities.

### 7.14 Anticoagulant activities

To investigate the anticoagulant potential of *Ferulago carduchorum*, Golfakhrabadi and co-researchers isolated the coumarins suberosin 38 and suberenol 39 (Fig. 23) from its aerial parts and evaluated their effects on prothrombin time (PT) in rats. Both compounds significantly prolonged PT at 3 and 6 mg kg^−1^ (*P* < 0.05), with suberosin at 6 mg kg^−1^ producing the greatest effect (17.4 s at 6 mg kg^−1^). The safety of the parent *F. carduchorum* extract was assessed in parallel: its acute LD_50_ exceeded 2000 mg kg^−1^, while daily oral administration of 250, 500, or 1000 mg kg^−1^ for 30 days produced no significant changes in haematological or biochemical parameters compared with controls (*P* > 0.05).^102^

**Fig. 23:**
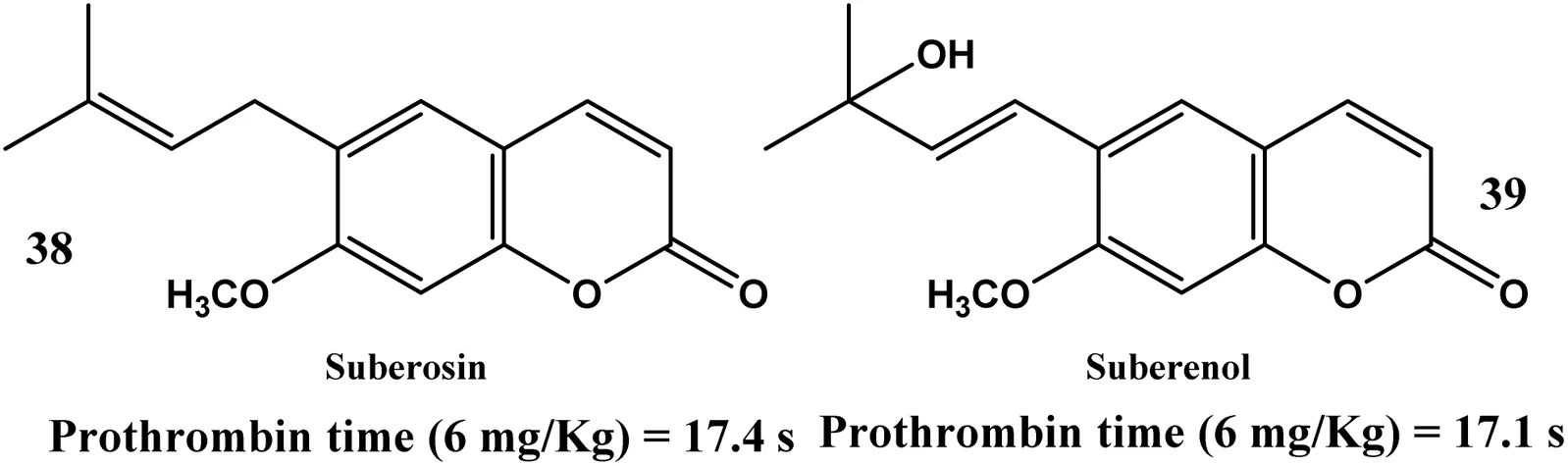
Anticoagulant suberosin and suberenol.

## 8 Limitations of study

This review is limited by the heterogeneity of reported synthetic methods, reaction conditions, and biological evaluation protocols, which makes direct comparison of coumarin derivatives across studies challenging. In addition, the biological activities of coumarin derivatives have been evaluated predominantly through *in vitro* and *in vivo* assays. However, for many of these derivatives, information regarding their specific molecular mechanism of action, pharmacokinetics, toxicity, and overall safety profiles is not consistently reported.

## 9 Conclusion

Coumarins represent a versatile chemical scaffold with demonstrated potential across anticoagulant, antimicrobial, antiparasitic, antiviral, anticancer, anti-inflammatory, neurological, metabolic, and enzyme-inhibitory applications. The literature reviewed indicates that their therapeutic potential is strongly influenced by the substitution pattern and functionalization of the coumarin core, with C3-, C4-, C6-, and C7-substituted derivatives providing particularly useful opportunities for tuning biological activity. Among the synthetic approaches, multicomponent and one-pot strategies, catalytic annulation/cyclization reactions, and other step-economical methods appear particularly attractive for generating structurally diverse coumarin libraries while reducing reaction steps, solvent consumption, and purification requirements. However, greater emphasis is still needed on green and scalable synthesis, including the use of safer solvents, recyclable catalysts, lower-energy conditions, and readily available starting materials, to facilitate translation from laboratory-scale discovery to larger-scale production.

The pharmacological literature also reveals substantial differences in the maturity of structure–activity relationship (SAR) data across therapeutic areas. The 4-hydroxycoumarin anticoagulants represent the most established example, with well-defined structural requirements and clinically validated mechanisms. In contrast, many of the reported antimicrobial, antitubercular, antiparasitic, antiviral – including *anti*-SARS-CoV-2 and neuropharmacological derivatives remain at an early discovery stage, with activity often reported for a limited number of analogues and without systematic SAR, target validation, or comprehensive selectivity and toxicity profiling. In particular, the antiviral and antitubercular areas would benefit from expanded analogue series, quantitative SAR analyses, experimentally validated molecular targets, and comparative evaluation against established therapeutic agents. Similarly, several reported activities rely predominantly on *in vitro* assays or computational predictions, highlighting the need for orthogonal biological validation.

Future investigations should therefore move beyond the identification of isolated active coumarin derivatives towards systematic scaffold optimization and mechanism-driven drug discovery. Priority should be given to integrating efficient and sustainable synthetic methodologies with systematic SAR, target-based biochemical and cellular assays, ADME/pharmacokinetic and toxicity evaluation, and appropriate *in vivo* models. Such integrated approaches will help distinguish genuinely promising coumarin-based leads from preliminary hits and facilitate the development of coumarin derivatives with improved potency, selectivity, safety, and translational potential.

## Author contributions

OOA – conceptualization and supervision; GOO & ESS wrote the chemistry aspect and prepared the draft; GOO, MLE & OYA wrote the pharmacology aspect and prepared the draft. All authors have given approval to the journal version of the review.

## Conflicts of interest

The authors have declared no conflicts of interest.

## Data Availability

No primary research results, software or code have been included and no new data were generated or analysed as part of this review.
